# Half-Metallic Transport and Spin-Polarized Tunneling through the van der Waals Ferromagnet Fe_4_GeTe_2_

**DOI:** 10.1021/acs.nanolett.4c01479

**Published:** 2024-07-22

**Authors:** Anita Halder, Declan Nell, Antik Sihi, Akash Bajaj, Stefano Sanvito, Andrea Droghetti

**Affiliations:** † School of Physics and CRANN, Trinity College, Dublin 2, Ireland; ‡ Department of Physics, SRM University − AP, Amaravati 522 502, Andhra Pradesh, India; § Institute for Superconducting and Other Innovative Materials for Devices, Italian National Research Council (CNR-SPIN), G. D’Annunzio University, Chieti 66100, Italy

**Keywords:** Spin transport, Tunnel magnetoresistance, van der Waals magnetic materials, Density functional theory, Nonequilibrium Green’s functions

## Abstract

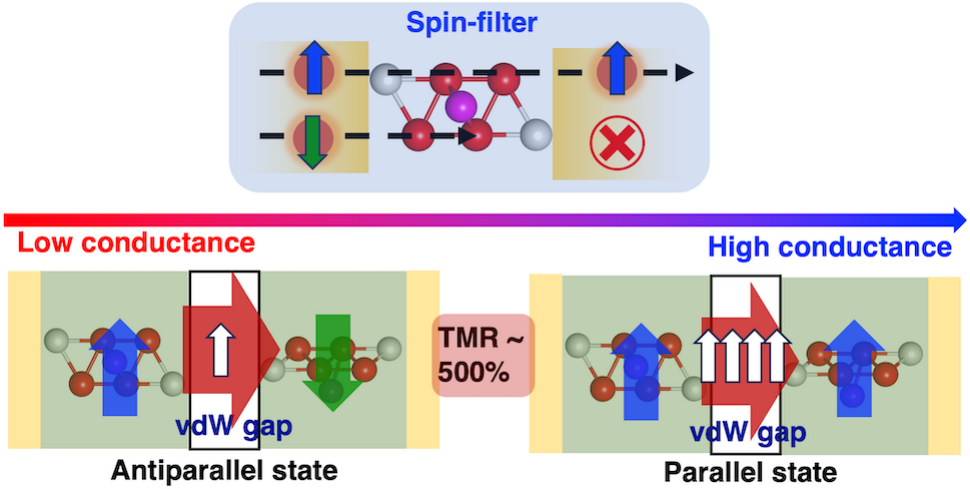

We examine the coherent spin-dependent transport properties of the van der Waals (vdW) ferromagnet Fe_4_GeTe_2_ using density functional theory combined with the nonequilibrium Green’s function method. Our findings reveal that the conductance perpendicular to the layers is half-metallic, meaning that it is almost entirely spin-polarized. This property persists from the bulk to a single layer, even under significant bias voltages and with spin–orbit coupling. Additionally, using dynamical mean field theory for quantum transport, we demonstrate that electron correlations are important for magnetic properties but minimally impact the conductance, preserving almost perfect spin-polarization. Motivated by these results, we then study the tunnel magnetoresistance (TMR) in a magnetic tunnel junction consisting of two Fe_4_GeTe_2_ layers with the vdW gap acting as an insulating barrier. We predict a TMR ratio of ∼500%, which can be further enhanced by increasing the number of Fe_4_GeTe_2_ layers in the junction.

Magnetic tunnel junctions (MTJs), which consist of two metallic ferromagnets separated by a thin insulating barrier, display the tunnel magnetoresistance (TMR) effect, that is, a variation in the charge current when the magnetizations of the two ferromagnets change their relative alignments.^1−5^ Recently, the discovery of magnetism in van der Waals (vdW) materials^6,7^ has created new opportunities for realizing MTJs. A significant magnetoresistance was initially reported in devices with the insulating material CrI_3_ sandwiched between graphite layers,^8,9^ while currently most studies focus on the Fe_n_GeTe_2_ (FGT) (*n* = 3–5) family of vdW metallic ferromagnets.^10^ Various FGT-based MTJs incorporating h-BN,^11,12^ graphite,^13^ MoS_2_,^14^ InSe,^15^ GaSe,^16^ or WSe_2_,^17^ between Fe_3_GeTe_2_ electrodes have been experimentally realized, recording a maximum TMR ratio of 300%.^11^ At the same time, first-principles calculations for similar systems^19−21^ predicted TMR ratios exceeding 1000% or multiple nonvolatile resistance states.

Among the FGT compounds, Fe_3_GeTe_2_ was the first reported in an MTJ^12^ and is the most studied. However, it has the lowest *T*_C_ (220 K) and requires gating to achieve room-temperature ferromagnetism in few-layer samples.^22^ Fe_5_GeTe_2_ has the highest *T*_C_ (310 K) but exhibits a complex magnetic behavior that remains unclear from both theoretical^23^ and experimental^24^ perspectives. Additionally, it is difficult to exfoliate,^25^ despite recent successful reports.^26^ Fe_4_GeTe_2_ (F4GT) has an intermediate *T*_C_ of 280 K and is easily exfoliated, maintaining ferromagnetism in few-layer samples.^10^ Recent experimental studies have shown its potential for generating highly spin-polarized currents,^27^ but its transport properties have not been systematically studied to date.

In this letter, we employ density functional theory (DFT),^28^ combined with the nonequilibrium Green’s function (NEGF) technique,^29^ to investigate the spin-dependent coherent transport properties of F4GT from first principles. Our findings reveal that the coherent transport perpendicular to the layers exhibits nearly half-metallic character, meaning that the charge current is almost perfectly spin-polarized. This characteristic persists from bulk to monolayer, even under significant bias and in the presence of spin–orbit coupling (SOC), making an F4GT layer an almost ideal spin-filter. Additionally, we analyze the impact of electron correlations, neglected in previous theoretical transport studies of FGT and similar vdW magnets despite their importance for the magnetic properties of these materials.^23,30^ By using a recently developed extension of dynamical mean field theory (DMFT) for quantum transport,^31^ we show that the combined effect of static and dynamical correlations preserves the conductance’s spin-polarization. Finally, we study a MTJ formed by two F4GT layers with the vdW gap acting as an insulating barrier, where we predict a high TMR ratio.

The DFT-NEGF transport calculations are performed by using the Smeagol code,^32−34^ which interfaces the implementation of the NEGF technique with the Siesta DFT package.^35^ We consider the Perdew–Burke–Ernzerhof generalized gradient approximation (GGA)^36^ for the exchange-correlation functional in all calculations, unless stated otherwise. The computational details are provided in Section S1 of the Supporting Information. The studied systems are shown in Figure 1a,b and consist of a central region and two semi-infinite leads. A finite bias voltage, *V*, is applied across the central region by shifting the chemical potentials of the leads as μ_L/R_ = *E*_F_ ± *eV*/2, where *E*_F_ is the Fermi energy and *e* the electron charge. Both zero- and finite-bias calculations are performed self-consistently.

**Figure 1:**
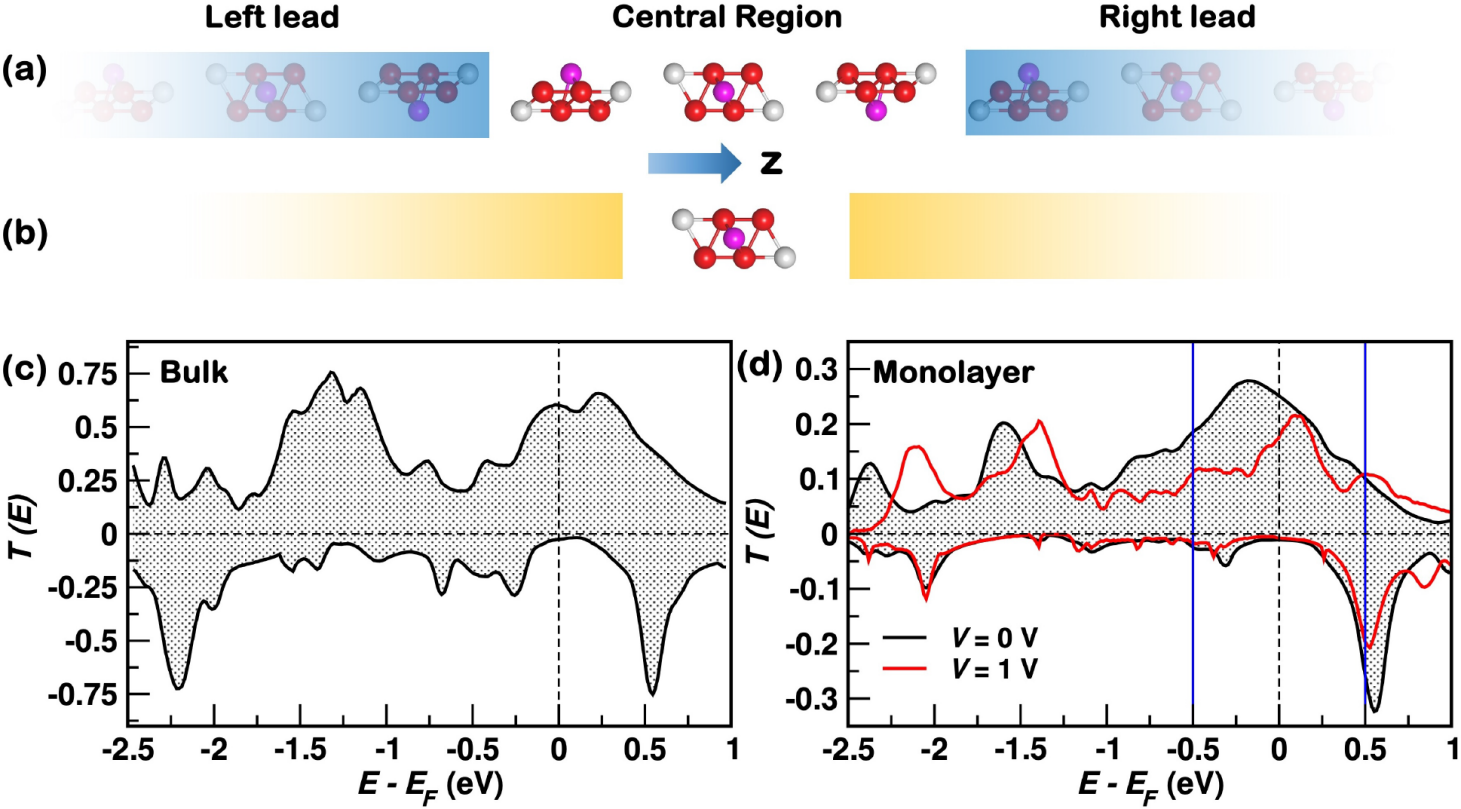
Coherent transport through F4GT. (a) Device made of an infinite number of F4GT layers. Red, gray, and magenta spheres represent Fe, Te, and Ge, respectively. (b) A device comprising an F4GT layer between model leads, represented as semi-infinite yellow rectangles. (c) Spin-up (positive) and spin-down (negative) transmission coefficient at zero-bias for the device in (a). (d) Spin-up (positive) and spin-down (negative) transmission coefficients at zero-bias and at a *V* = 1 V for the monolayer device in (b). The vertical blue lines delimit the bias window between *E*_F_ – *eV*/2 and *E*_F_ + *eV*/2 for *V* = 1 V.

We initially assume a two-spin-fluid picture^37^ for coherent charge transport and perform spin-collinear calculations, following common practice in the study of MTJs.^3^ Under this assumption, the two spin channels conduct in parallel without mixing, and the charge current for spin σ (=*↑*, *↓*) is defined as^29^
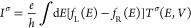
1where *h* is Planck’s constant, *f*_L(R)_(*E*) = [1 + *e*^β(*E*–μ_L(R)_)^]^−1^ is the Fermi function of the left (right) lead, and β is the inverse temperature. The spin-, energy-, and bias-dependent transmission coefficient, *T*^σ^(*E*, *V*), is calculated through the Fisher-Lee formula.^38^ According to eq 1, the transport is determined by the coherent transmission of spin-up and -down electrons from one lead, through the central region, to the other lead. The transmission coefficient depends on *V* because the electronic states may shift in energy under the applied bias (see, for instance, refs (65 and 39)). Notably, *I*^σ^ in eq 1 is approximately equal to the area under the transmission coefficient-vs-energy curve inside the energy interval [*E*_F_ – *eV*/2, *E*_F_ + *eV*/2], known as bias window. In the linear-response limit and at zero temperature, the expansion of eq 1 returns the conductance of each spin channel through the Landauer-Büttiker formula, *G*^σ^ = *G*_0_*T*^σ^(*E*_F_, *V* = 0),^40−42^ with  denoting the quantum of conductance. In the following, the dependence of the transmission coefficient on *V* is neglected to keep a concise notation.

We start by calculating the transport properties of a device that consists of an infinite number of F4GT layers with ABC stacking,^10^ as shown in Figure 1(a). We consider the transport perpendicular to the layers, that is, along the *z* direction, with periodic boundary conditions in the *xy* plane. More details can be found in Section S2 of the Supporting Information. The zero-bias spin-resolved transmission coefficient is plotted in Figure 1(c). *T*^*↑*^(*E*) exhibits a prominent peak, whereas *T*^*↓*^(*E*) is gapped around *E*_F_. Hence, according to the Landauer-Büttiker formula, the linear-response spin-down conductance is negligible compared to the spin-up conductance, implying half-metallic transport.

To obtain a better understanding of our result, in Figure 2 we plot the F4GT spin-resolved band structure, where the blue, green, and red bands have predominant amplitude over the Fe 3d_*xz*_, 3d_*yz*_, and 3d_*z*^2^_ orbitals, respectively. We observe both spin-up (majority) and spin-down (minority) bands, cutting *E*_F_ at several points in the Brillouin zone. However, the situation is different when we restrict the analysis to the Γ-*A* direction only, where the momentum has components (*ℏk*_*x*_, *ℏk*_*y*_) = 0 and *ℏk*_*z*_ > 0; i.e., the momentum is perpendicular to the layers. In the majority channel, there is a band with d_*z*^2^_ character crossing *E*_F_, whereas the minority channel has a band gap with a minimum *E*_g_ ∼ 0.2 eV at *A*, along with dispersionless valence bands. Thus, only majority Bloch states can carry current in the perpendicular direction within the linear-response limit, leading to half-metallic transport behavior. Notably, the density of the states (DOS) along the Γ-*A* direction (displayed beside the band structure) is typical of a half-metal.

**Figure 2:**
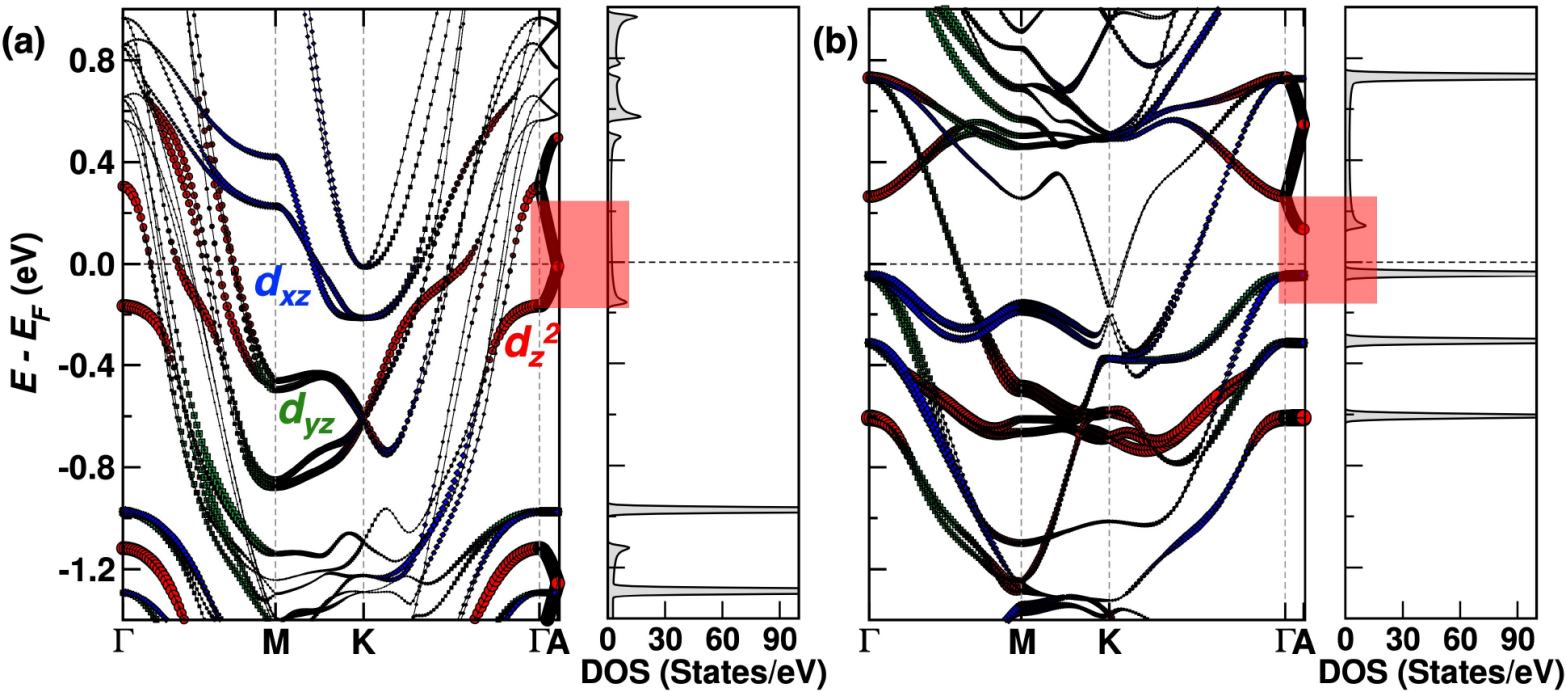
Band structure of bulk F4GT: (a) spin-up bands and (b) spin-down bands. The width and color of the bands indicate the orbital character. Bands with Fe 3d_*xz*_, 3d_*yz*_, and 3d_*z*^2^_ orbital character are blue, green, and red, respectively. On the right-hand side of each band structure, we show the density of the states with momentum *ℏk*_*z*_ > 0 along the Γ-*A* direction in the Brillouin zone. The energy region around *E*_F_ is highlighted in red.

The calculated conductance’s spin-polarization, defined as , is as high as 0.92, though not perfect. This is because, although there are no minority bands at *E*_F_ along Γ-*A*, there are a few other minority states with nonzero transverse momentum, as revealed in the plot of the Fermi surface and momentum-resolved transmission coefficient in Section 10-A of the Supporting Information. Despite this, F4GT outperforms the related compounds Fe_3_GeTe_2_ and Fe_5_GeTe_2_ in terms of spin-transport properties. Fe_3_GeTe_2_ has some transport due to minority states at the center of the Fermi surface,^20^ reducing the spin-polarization, while Fe_5_GeTe_2_ has a band gap along Γ-*A* in both spin channels (see the Supporting Information of ref (19)), limiting both spin-up and -down electron transport.

The properties of F4GT can be further understood by focusing on the monolayer limit and systematically assessing various factors that can affect spin transport. Therefore, we now investigate the device of Figure 1(b), featuring one F4GT layer between two “model” leads (see Section S2–C of the Supporting Information). The transmission coefficient for this device, shown in Figure 1(d), resembles that of bulk F4GT, displaying a prominent peak (gap) in the spin-up (down) channel around *E*_F_. *G*^*↑*^ is ∼0.25*G*_0_, while *G*^*↓*^ is much smaller, resulting in SP = 0.92, which is the same as the bulk value. The monolayer effectively acts as an almost ideal spin-filter.

The origin of the half-metallic transport behavior in the monolayer device is analyzed in terms of the DOS and Fermi surface in Sections S6 and S10–B of the Supporting Information. Specifically, we find that in the spin-up channel, a strong hybridization of the Fe 3d_*z*^2^_ and Te 5p_*z*_ orbitals results in a delocalized, and therefore highly conductive, state at *E*_F_. Eventually, this delocalized state evolves into the dispersive spin-up band observed along the Γ-*A* direction in Figure 2 as the F4GT structure transitions from a monolayer to bulk. In contrast, in the spin-down channel, the delocalized state is at *E* – *E*_F_ ≈ 0.5 eV, and there are no states at *E*_F_ for conduction. Notably, this spin asymmetry persists even with doping, introduced, for example, by changing the work function of the leads, as discussed in Section S11–C of the Supporting Information. The nearly half-metallic behavior remains a robust characteristic of the system.

The study of the F4GT-monolayer device can be extended beyond the linear-response limit by performing finite-bias calculations. The electronic structure is found to change with the bias, *V*, as explained in Section S7 of the Supporting Information. However, the transmission coefficient [red curve in Figure 1(d)] remains half-metallic, with a spin-down gap at *E*_F_. The charge and spin currents, respectively, defined as *I* = *I*^*↑*^ + *I*^*↓*^ and *I*^s^ = *I*^*↑*^ – *I*^*↓*^, are plotted in Figure 3 as a function of *V*. These curves are understood by recalling that *I*^*↑*(*↓*)^ is approximately equal to the area under the spin-up (down) transmission curve inside the bias window [see eq 1], which is delimited by the blue bars in Figure 1(d). At low biases (*V* ≲ 0.3 V), *I*^*↑*^ dominates while *I*^*↓*^ is negligible because of the half-metallic character of the transmission coefficient. Thus, *I* (solid curve) and *I*^*s*^ (dotted curve) are identical, and the current spin-polarization, *I*^s^/*I*, is about 1. In contrast, at high biases (*V* ≳ 0.6 V), *I*^*↓*^ starts increasing with *V* as the spin-down gap’s edges enter the bias window [see Figure 1(d)]. The electrons from the spin-down conduction states then contribute to the transport in parallel with the spin-up electrons, reducing *I*^*s*^, and the system does not show half-metallic conductance anymore. Nonetheless, the spin-polarization remains as large as ∼0.7 at *V* = 1 V. Thus, an F4GT monolayer acts as an effective spin-filter even up to high biases.

**Figure 3:**
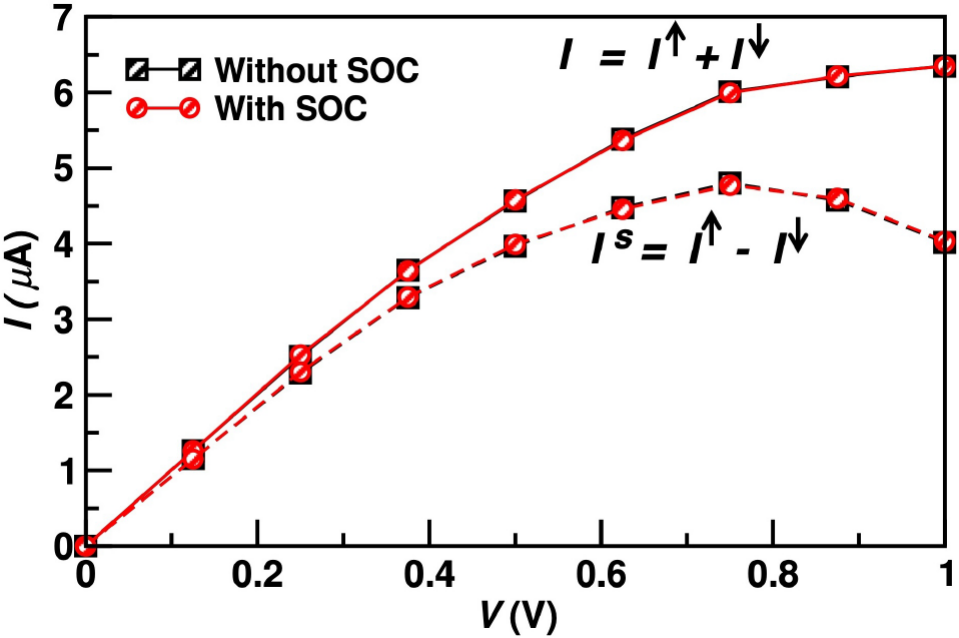
Results of the finite-bias calculations for the monolayer device. Charge and spin currents, *I* and *I*^s^, as a function of bias voltage, *V*. Black (red) points are the results obtained without (with) SOC.

To further check the robustness of our predictions, we now introduce the SOC,^43^ neglected up to this point. SOC provides a mechanism for spin-mixing, invalidating the two-spin-fluid picture. In many materials, it is known to degrade the spin-polarization.^44,45^ In F4GT, its effect on the electronic properties is significant, as seen in the bulk band structure with SOC [Figure S3 in the Supporting Information]. Nevertheless, the band crossing the Fermi level along Γ-*A* maintains a well-defined spin character, preserving the spin-polarization.

For the monolayer case, the calculations with SOC can be further extended to finite-bias. The spin-resolved currents are no longer defined, but we use the so-called “bond current” approach^46^ to derive a general definition of the spin current, *I*_s_, valid also in the presence of SOC.^47,48^ The results are presented as red circles in Figure 3. They appear indistinguishable from those obtained without SOC (black squares), confirming that spin-mixing is negligible in the transport through our system and the predictions based on the two-spin-fluid picture are reliable.

We now analyze electron correlation effects beyond the GGA. In general, electron correlations impact transport through ferromagnetic metallic layers by inducing an energy shift of the conductive electronic states.^31,49^ Furthermore, in half-metals, dynamical correlations may also give rise to non-quasiparticle peaks in the insulating spin channel,^50^ thus quenching the perfect spin-polarization. In the case of the FGT compounds, experimental observations suggest a competition between itinerant and localized electrons,^30^ and theoretical studies^23^ report that dynamical correlation is essential to accurately describe magnetic properties. FGT compounds have therefore been regarded as moderately correlated materials.

We carry out calculations for the monolayer device by using DFT+U^51−52^ and DFT+DMFT^53,54^ with the implementation described in refs (31, 55, and 56). In DFT+U, an effective Hubbard-like *U* interaction for the Fe 3d orbitals is added to the GGA exchange-correlation functional and is treated at the static mean-field level. In contrast, DFT+DMFT accounts also for dynamical correlation (albeit local in space) via an energy-dependent self-energy.^53,54^ By comparing DFT+U and DFT+DMFT results, we gain insights into the relative importance of static versus dynamical correlations. We consider only the zero-bias limit, restoring the two-spin-fluid picture, which we have just shown to be appropriate for our system.

The DFT+U and DFT+DMFT transmission coefficients are presented in Figure 4 (the DOS is shown in Section S8 of the Supporting Information). Static correlation, as described by DFT+U, enhances the spin splitting of the Fe 3d_*z*^2^_ states compared to DFT (see Section S8-A of the Supporting Information). As a result, the main peak of *T*^*↑*^(*E*) moves from *E*_F_ to lower energies by about 0.5 eV, and the linear-response conductance is reduced by more than half with respect to the DFT value. Conversely, in *T*^*↓*^(*E*), the gap’s center shifts from *E*_F_ toward higher energies, so that the valence states cross *E*_F_, increasing the spin-down conductance. Overall, DFT+U reduces the linear-response conductance’s spin-polarization to ∼0.5.

**Figure 4:**
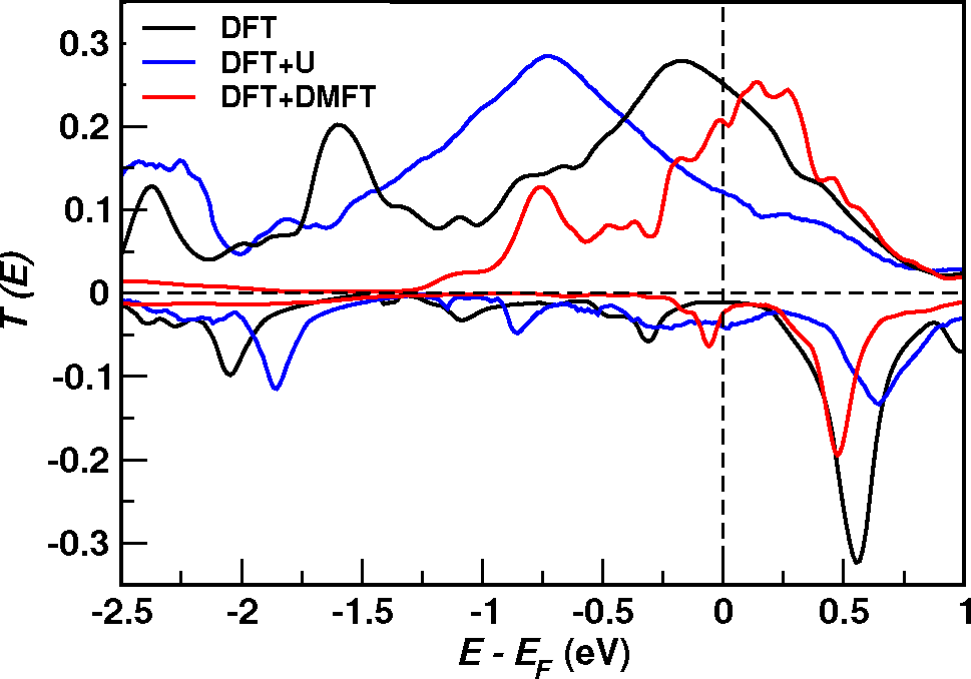
Zero-bias transmission coefficient calculated by using DFT (black curve), DFT+U (blue curve), and DFT+DMFT (red curve).

The inclusion of dynamical correlation by means of DMFT redistributes the Fe 3d states in energy, counterbalancing the effect of static correlation and reducing the spin splitting (see Section S8–C in the Supporting Information). On the one hand, the main peak in the spin-up transmission is narrowed but, once again, centered near *E*_F_. On the other hand, the spin-down transmission remains insulating, although the size of transport gap is reduced compared to the DFT one. We find no non-quasiparticle peaks. The change of the transmission coefficient from DFT(+U) to DFT+DMFT calculations can be ascribed uniquely to the energy shift and the finite lifetime of the 3d quasi-particle states. Overall, these calculations indicate that although electron correlations beyond DFT are important in F4GT, the combined effect of static and dynamical contributions preserves the almost perfect spin-filter character predicted by DFT.

The transport properties of F4GT can eventually be exploited in MTJs. This possibility is explored by considering the idealized device in Figure 5(a). The central region, attached to the same model leads used before, comprises two F4GT layers (L1 and L2), separated by the vdW gap that serves as the insulating barrier. The device can be set in two configurations with the magnetization vectors of the two F4GT layers being either parallel (P) or antiparallel (AP) to each other. The calculations are carried out by using spin-collinear DFT, which captures the key transport features, as explained before. The charge current as a function of the applied bias voltage, *V*, for the two configurations is displayed in Figure 5(b). At low bias, the P current, *I*_P_, is significantly larger than the AP current, *I*_AP_. In contrast, with increasing *V*, *I*_P_ tends to saturate, while *I*_AP_ sharply increases. As a result, the TMR ratio, defined as (*I*_P_ – *I*_AP_)/*I*_AP_, is as large as 460% at low bias (*V* < 0.15 V) and then drops with *V*, becoming about 50% at 0.75 V. Notably, the large zero-bias TMR remains rather unaffected by (unstructured) disorder, and is, in fact, even slightly enhanced, as discussed in Section S11-A of the Supporting Information, demonstrating the robustness of the system’s properties.

**Figure 5:**
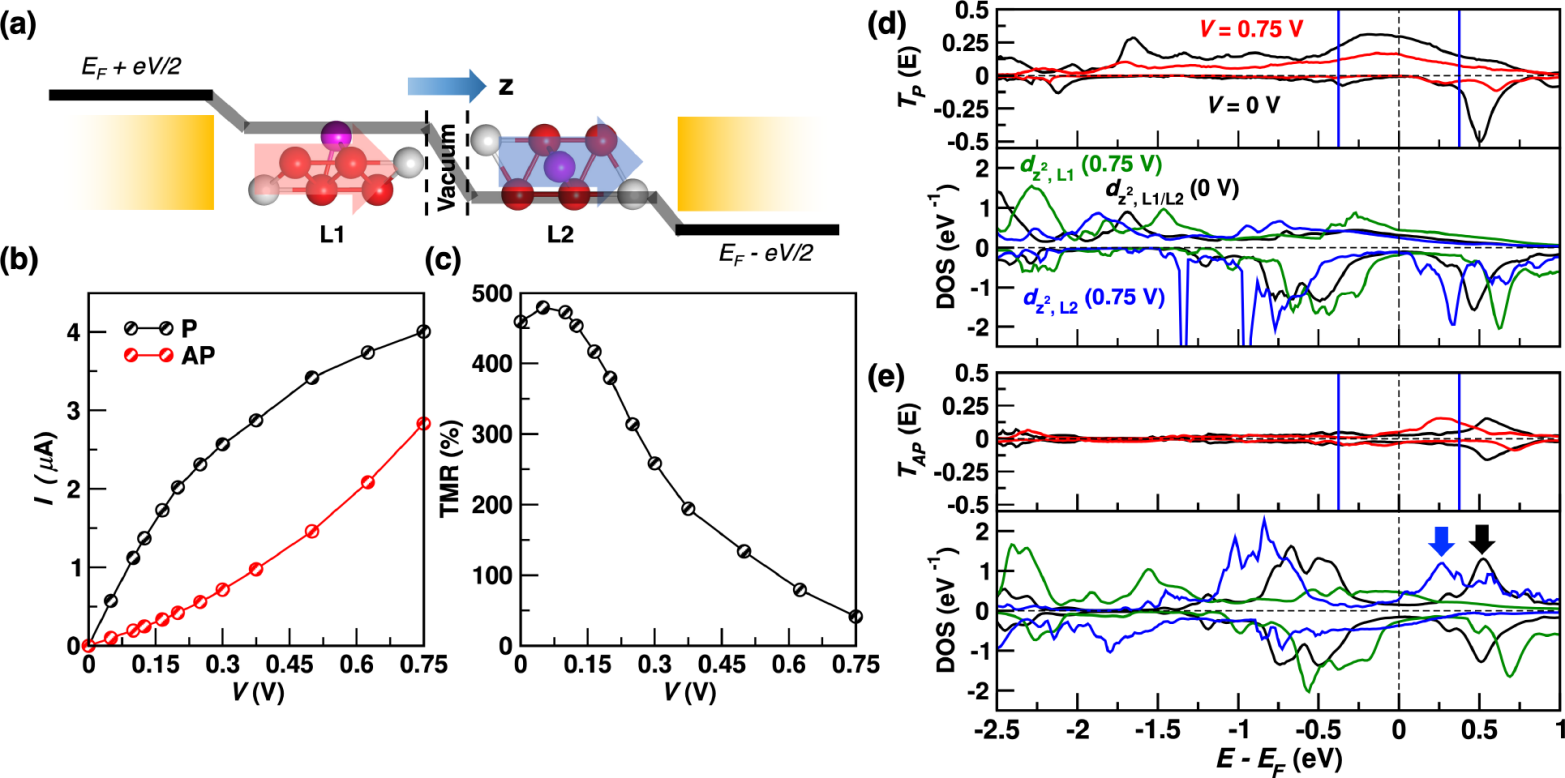
Results for the F4GT-based MTJ. (a) The MTJ consists of two F4GT layers, denoted as L1 and L2, sandwiched between model leads, which are represented as semi-infinite yellow rectangles. The transport direction is along the *z* Cartesian axis. The magnetization vectors of the two F4GT layers in the P configuration are pictured as thick red and blue arrows for L1 and L2, respectively. The electrostatic potential drop in the central region is also shown schematically as a black thick line. It drops linearly across the vdW gap, while it remains nearly constant inside the F4GT layers. (b) The current–voltage characteristic curve for the P and AP configurations. (c) TMR ratio as a function of bias voltage. (d) The transmission coefficient (upper panel) and the DOS projected over Fe 3d_*z*^2^_ orbitals of L1 and L2 (lower panel) for the P configuration at zero bias and at *V* = 0.75 V. (e) Same as (d) for the AP configuration. Note that the spin-up and spin-down DOS do not look identical in the AP configuration because the system is not exactly inversion-symmetric with respect to the center of the device. The black (blue) arrow indicates the position of the spin-up conduction states of L2 at zero bias (*V* = 0.75 V).

At zero-bias, the TMR is understood through the standard Julliere’s phenomenological description.^3^ We assume transport from left to right so that the left F4GT layer (L1) filters spin-up electrons, which are then detected by the right layer (L2). In the P configuration, since L2 is metallic in the spin-up channel, spin-up electrons are transmitted through. In contrast, in the AP configuration, the L2 spin-up channel becomes insulating and transport is greatly suppressed. As a result, the TMR is large. Quantitatively, the effect is analyzed in terms of the transmission coefficients in Figures 5(d) and 5(e) for the P and AP configurations, respectively (also see the momentum-resolved results in Section S10–C in the Supporting Information). *T*_P_^σ^(*E*) appears similar to its counterpart for the monolayer device, and the conductance *G*^*↑*^ is as large as ∼0.25 *G*_0_. In contrast, *T*_AP_^σ^(*E*) is approximately given by the convolution of the spin-up (metallic-like) and spin-down (insulating-like) transmissions of the monolayer, as expected based on the standard model of MTJs.^34,60^ As such, it nearly vanishes at *E*_F_.

At finite-bias, *I*_P_ and *I*_AP_, and therefore the TMR ratio, depend on the change of the energy alignment between Fe states of L1 and L2. Since the electrostatic potential predominantly drops across the vdW gap between the two F4GT layers, as schematically drawn in Figure 5(a), the states in L1 (L2) are pinned to the left (right) lead and experience an upward (downward) energy shift with increasing *V*. In the P configuration [Figure 5(d)], the Fe 3d_*z*^2^_ DOS of L1 and L2 become misaligned with *V*, leading to a reduced electronic overlap through the vdW barrier and to a partial suppression of the transmission coefficient. Conversely, in the AP configuration [Figure 5(e)], the behavior is somewhat opposite. The spin-up channel of L2 is insulating preventing transport at low bias. Yet, with increasing *V*, the L2 spin-down conduction states [indicated by the arrows in the bottom panel of Figure 5(e)] move down in energy until they eventually enter the bias window. When that happens, the electrons filtered by L1 can be transmitted though L2, leading to a sharp *I*_AP_ increase and therefore to a TMR ratio drop.

Interestingly, a recent quantum transport study^62^ predicted a TMR ratio of just ∼24% at zero-bias in a MTJ made of two F4GT layers. However, in that case the transport was in-plane, while, as shown here, the large spin-polarization is characteristic only of the perpendicular direction. Notably, in this perpendicular case, the zero-bias TMR ratio can be further enhanced by increasing the number of F4GT layers acting as spin-filters. For example, calculations for a three-layer device, presented in Section S9 of the Supporting Information, give a huge TMR ratio exceeding 1200%. This value is comparable to the one predicted in Fe(001)/MgO MTJs^63^ used in technological applications.

In practice, operating F4GT-based MTJs in experiments requires the capability of switching the magnetization of a layer independently from that of the others. This can be achieved, for example, by substituting some of the perfect F4GT layers with slightly off-stoichiometric compounds, such as Fe_4–x_GeTe_2_,^64^ characterized by a different coercive field. Alternatively, one may place the spin-filter and detector layers in contact with leads made of different heavy metals, thus tuning their relative magnetic anisotropy by proximity.

In summary, our first-principles calculations have revealed the spin-filtering capability of the vdW ferromagnet F4GT along the perpendicular direction, demonstrating nearly half-metallic conduction. This property remains robust even up to relatively large bias voltages and in the presence of SOC, doping, and electron correlations. F4GT therefore represents an extraordinary material for spintronics.

## Supporting Information Available

Computational details; F4GT structures and geometry optimizations; Band structure with SOC; Complex band structure of bulk F4GT; DOS and orbital occupations; PDOS analysis for the F4GT monolayer; Electronic structure of the F4GT monolayer at finite bias; Electronic structure of the F4GT monolayer predicted by DFT+U and DFT+DMFT calculations; Results for a MTJ comprising three F4GT layers; Fermi surfaces and zero-bias **k**-resolved transmission coefficients; Analysis of the effect of disorder, strain, and work function changes on the spin-transport properties of the F4GT devices (PDF)
