# Supplementary material for: Half-Metallic Transport and Spin-Polarized Tunneling through the van der Waals Ferromagnet Fe4GeTe2
Source: Nano Lett. 2024 Jul 22;24(30):9221–8. doi: 10.1021/acs.nanolett.4c01479 (PMC11299226; doi:10.1021/acs.nanolett.4c01479)
Supplement: Supplementary file 1 — nl4c01479_si_001.pdf [file nl4c01479_si_001.pdf]

Supporting Information
Half-Metallic Transport and Spin-Polarized Tunneling through the van der Waals Ferromagnet Fe_4GeTe_2

Anita Halder,[1,2] Declan Nell,[1] Antik Sihi,[1] Akash Bajaj,[1] Stefano Sanvito,[1] and Andrea Droghetti[1,3,*]

[1]*School of Physics and CRANN, Trinity College, Dublin 2, Ireland*
[2]*Department of Physics, SRM University – AP, Amaravati 522 502, Andhra Pradesh, India*
[3]*Institute for Superconducting and Other Innovative Materials for Devices, Italian National Research Council (CNR-SPIN), G. D'Annunzio University, Chieti 66100, Italy*

S1. COMPUTATIONAL DETAILS

A. DFT calculations with SIESTA

Spin-collinear DFT calculations are performed using the PSML (pseudopotential markup language) compatible version of the SIESTA DFT package[1,2]. The Perdew-Burke-Ernzerhof (PBE)[3] generalized gradient approximation (GGA) is assumed for the exchange-correlation density functional, eventually also including the van der Waals (vdW) interaction through the D3 correction[4] for the geometry optimizations of the bulk and multilayer systems. Core electrons are treated using norm-conserving Troullier-Martins pseudopotentials[5,6]. The *spd* valence electrons are expanded using the numerical atomic orbital basis set of double-ζ quality, while additional polarization functions are incorporated into the 4*s* Fe orbitals[7,8]. The cutoff radii of the basis orbitals are taken from Ref. [9].

A $12 \times 12 \times 8(1)$ Γ-point centered Monkhorst-Pack **k**-mesh is used for bulk (monolayer) F4GT. The plane-wave cutoff corresponding to the resolution of the real-space density grid is set to 600 Ry. The atomic coordinates are optimized until all atomic forces are less than 0.05 eV/Å.

The orbital-resolved band structure of bulk F4GT is plotted by means of the `fat` and `eigfat2plot` utilities available within SIESTA.

The Fermi surfaces are obtained using energy dispersion data from band structure calculations, employing the SIESTA utility `eig2bxsf` to format the data correctly. XCRYSDEN[10] is used to generate the energy isosurfaces of the Fermi surfaces and for their visualization. These surfaces are then projected onto a 2D plane perpendicular to the transport direction. For the monolayer Fermi surface, a single layer of F4GT is consider within a supercell, with a 30 Å vacuum gap separating periodic images in the perpendicular direction.

The calculations including spin-orbit coupling (SOC) are carried out by using a locally modified version of SIESTA. The on-site approximation of Ref. [11] is assumed for the spin-orbit matrix elements. All computational parameters are the same as in calculations without SOC.

B. DFT calculations with VASP

Additional DFT calculations are carried out by using DFT as implemented in the Vienna *Ab-initio* Simulation Package (VASP)[12], which has been found to be more accurate than SIESTA for total energies and forces. The PBE GGA[3] exchange-correlation functional is used with the vdW corrections included by means of the D3 scheme[4]. The calculations are spin-polarized. A Γ-centered $30 \times 30 \times 6(1)$ **k**-mesh is employed for the bulk (monolayer), with a convergence criteria of 10^{-7} eV for the total energy. The structures are relaxed until all forces on the atoms are smaller than 10^{-3} eV/Å. The Gaussian smearing method is used with a kinetic-energy cutoff equal to 600 eV.

C. Quantum transport calculations

The quantum transport calculations are performed by using the NEGF method as implemented in the SMEAGOL code[13,14] which is interfaced with SIESTA. The basis set, pseudopotentials and exchange-correlation functional are the same as in the band structure calculations.

The studied systems consist of a central region attached to two semi-infinite leads, which are treated by means of self-energies[13]. Periodic boundary conditions are used in the x-y plane perpendicular to the transport direction z, and $\mathbf{k}_\parallel = (k_x, k_y)$ indicates the Bloch wavenumber in the transverse 2D Brillouin zone (BZ). The effect of applying a finite bias voltage V is simulated by shifting the leads relative chemical potentials in such a way that $\mu_L = E_\mathrm{F} + eV/2$ and $\mu_R = E_\mathrm{F} - eV/2$, where E_F is the Fermi level.

The NEGF equations are presented in Refs. [13–15]. They are solved for each $\mathbf{k}_\parallel$, and finally, the transmission coefficient in Eq. (1) of the paper is given by

$$T^\sigma(E) = \frac{1}{N_{\mathbf{k}_\parallel}} \sum_{\mathbf{k}_\parallel \in 2D\mathrm{BZ}} T^\sigma(E, \mathbf{k}_\parallel), \tag{1}$$

where $T^\sigma(E, \mathbf{k}_\parallel)$ is the $\mathbf{k}_\parallel$-resolved transmission coefficient and $N_{\mathbf{k}_\parallel}$ is the number of $\mathbf{k}_\parallel$-points.

The leads' self-energies are calculated by using a singular value decomposition-based algorithm[16]. Both

zero- and finite-bias calculations are performed self-consistently. The electrostatic Hartree potential, V_{H}, inside the central region is obtained by solving the Poisson equation. The difference, ΔV_{H}, between the Hartree potential at finite- and zero-bias gives the voltage drop across the central region.

The density matrix of the central region is calculated by splitting the integration of the lesser Green's function into the so-called equilibrium and nonequilibrium components[13]. The equilibrium component is obtained by performing the integration over a semicircular contour in the complex energy plane[13]. 16 poles are used in the Fermi distribution, and 16 energy points are used along both the semicircle and the imaginary line that form that contour. The nonequilibrium component is calculated by performing the integration over the real energy axis using 100 energy points inside the bias window.

The self-consistent density matrix is calculated with a 20×20 Γ-point centered Monkhorst-Pack $\mathbf{k}_{\|}$-mesh. This converged density matrix is then used as input for non-self consistent calculations, which employ a 100×100 $\mathbf{k}_{\|}$-mesh to obtain the transmission coefficient and the DOS of bulk F4GT, the monolayer device and the MTJs. The $\mathbf{k}_{\|}$-mesh is further refined in the calculations of the $\mathbf{k}_{\|}$-resolved transmission coefficients in Section S10 until smooth plots are achieved.

The bond current approach, as described in Refs. [17] and [18], is employed to calculate the charge and spin currents in the presence of SOC. In spin-collinear calculations, it was verified that the results obtained using the bond current approach match those obtained from Eq. (1) in the paper to within two significant digits.

D. DFT+DMFT calculations

DFT+DMFT calculations for the monolayer device are carried out by using SMEAGOL with the implementation described in Refs. [19] and [15]. The correlated subsystem, spanned by the Fe $3d$ orbitals, is downfolded from the device's central region by means of the scheme in Ref. [20]. General orbital-dependent Coulomb interaction parameters for the $3d$ orbitals within each Fe atom are considered. These are expressed in terms of Slater integrals F^0, F^2 and F^4 (Ref. [21]). The ratio F^4/F^2 is assumed to correspond to the atomic value ≈ 0.625 (Ref. [22]). The average U and J interaction parameters are given through the relations $U = F^0$ and $J = (F^2 + F^4)/14$.

Second-order perturbation theory in the electron-electron interaction is employed as the impurity solver for DMFT[19] allowing for the fast evaluation of the self-energy directly on the real energy axis with no need for any analytic continuation schemes. The first order contribution accounts for static (energy-independent) mean-field corrections to the DFT GGA Kohn-Sham Hamiltonian and is approximated with the U-potential by Dudarev *et al.*[23] For each Fe $3d$ orbital, λ, this has the form,

$$V^{\sigma}_{U,\lambda} = (U-J)(\frac{1}{2} - n^{\sigma}_{\lambda}), \tag{2}$$

where σ denotes the spin and n^{σ}_{λ} is the occupation. In practice, the use of only this first order correction coincides with performing DFT+U calculations. The potential U in Eq. (2) is positive (negative) for orbitals with less (more) than half-filling occupations n^{σ}_{λ}.

The second order contribution introduces dynamic correlation. The local Green's function is calculated by summing the retarded Green's function over a 20×20 Monkhorst-Pack $\mathbf{k}_{\|}$-mesh. The many-body self-energy is computed on an energy grid consisting of 3000 points, extending from -20 to 10 eV.

DFT+U calculations are performed evaluating the charge density self-consistently. In contrast, the DFT+DMFT calculations are not charge-self-consistent.

S2. F4GT STRUCTURES AND GEOMETRY OPTIMIZATIONS

A. Bulk F4GT

Bulk F4GT possesses a rhombohedral crystal structure with a *R-3m* space group, and experimental lattice parameters, $a = 4.04$ Å and $c = 29.08$ Å[24,25]. The primitive unit cell contains three layers separated along the c axis by a vdW gap. There are four Fe atoms, Fe1, Fe1', Fe2, and Fe2', in each layer (see the inset of Fig. S5). They occupy two inequivalent Wyckoff positions forming two pairs of Fe-Fe dumbbells directly bonded to the Te atoms. Fe1 and Fe2 are equivalent to Fe1' and Fe2'. The Ge atoms are positioned off the plane defined by the Fe-Te network. The layers are stacked in an ABC configuration along the c-axis, as shown in Fig. 1(a) in the paper. We assume a Cartesian frame of reference such that the c axis is parallel to the Cartesian z axis.

We use the experimental lattice parameters and optimize all atomic positions within the primitive unit cell using both VASP and SIESTA. The PBE functional[3], including van der Waals interactions via the D3 correction[4], is employed in both cases, as described in Section S1. The optimized atomic positions are reported in Table S1. The x and y atomic coordinates returned by VASP and SIESTA are nearly identical with a high accuracy (to within 10^{-5} Å). In contrast, there are slight differences in the z coordinates, but the maximum deviation is only about 0.02 Å. Since the total energies and forces can be converged to a much higher accuracy in the VASP than in the SIESTA calculations, we ultimately use the VASP optimized structures in our study.

The band structure in Fig. 2 of the paper is obtained using SIESTA while considering the VASP optimized atomic positions. On the other hand, in Fig. S1 here, we compare the band structure from the paper (black curve) with the SIESTA band structure calculated

	VASP			SIESTA		
species	x	y	z	x	y	z
Fe	0.000	0.000	9.056	0.000	0.000	9.063
Fe	0.000	0.000	20.024	0.000	0.000	20.016
Fe	2.020	1.166	18.749	0.000	0.000	18.757
Fe	2.020	1.166	0.637	2.020	1.166	0.630
Fe	0.000	2.332	28.442	0.0000	2.332	28.450
Fe	0.000	2.332	10.330	0.000	2.332	10.323
Fe	0.000	0.000	11.486	0.000	0.000	11.494
Fe	0.000	0.000	17.593	0.000	0.000	17.586
Fe	2.020	1.166	21.180	2.020	1.166	21.187
Fe	2.020	1.166	27.286	2.020	1.166	27.279
Fe	0.000	2.332	1.794	0.000	2.332	1.801
Fe	0.000	2.332	7.900	0.000	2.332	7.893
Ge	0.000	0.000	0.000	0.000	0.000	0.000
Ge	2.020	1.166	9.694	2.020	1.166	9.693
Ge	0.000	2.3325	19.386	0.000	2.332	19.387
Te	0.000	0.000	6.545	0.000	0.000	6.530
Te	0.000	0.000	22.535	0.000	0.000	22.550
Te	2.020	1.166	16.238	2.020	1.166	16.223
Te	2.020	1.166	3.149	2.020	1.166	3.164
Te	0.000	2.332	25.931	0.000	2.332	25.916
Te	0.000	2.332	12.842	0.000	2.332	12.857

TABLE S1. Atomic coordinates (in Å) of bulk F4GT optimized by using VASP and SIESTA.

using the SIESTA optimized atomic positions to assess potential differences. We observe that the two band structures nearly overlap for spin-up states, whereas there are some deviations for spin-down states. Nonetheless, along the Γ-A direction, which is relevant for transport, the spin-down band gap changes by only a few meV, which is negligible for the scope of our work.

The discrepancy between the two band structures depicted in Fig. S1 becomes more pronounced for spin-down states within the energy range from $E - E_\mathrm{F} \approx -0.4$ to -1.3 eV, particularly along the M-K and K-Γ directions. However, these directions are irrelevant for perpendicular transport, and furthermore, the bands are located at energies too low relative to the Fermi energy to contribute to the conductance.

Although differences in the band structures shown in Fig. S1 and in the atomic positions listed in Table S1 may be important for predicting electronic and magnetic properties of bulk F4GT, they do not impact the predicted transport properties.

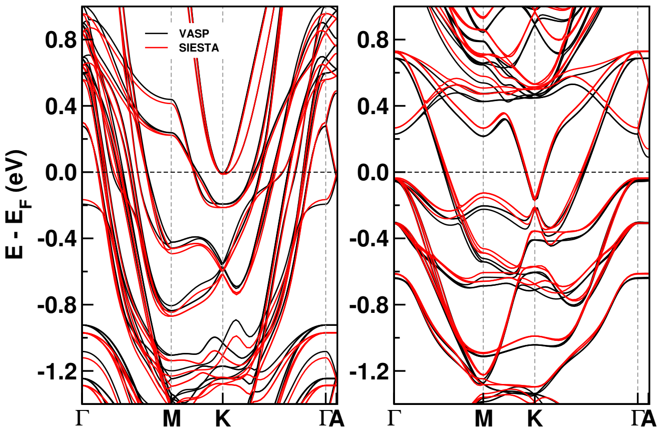

FIG. S1. Band structures calculated by using SIESTA for the VASP and SIESTA optimized geometries (black and red curves, respectively).

B. Monolayer and bilayer

The structures of the F4GT monolayer and bilayer are optimized by using VASP, following the same computational details as in Ref. [26], which also employed VASP. Our results are in perfect agreement with those reported in that paper. The obtained lattice constant of the monolayer and bilayer are respectively $a = 3.92$ Å and 3.93 Å. The bilayer is assumed to have AB stacking and the calculated vdW gap is 3.03 Å.

C. Model device

Since our primary goal is to explore the fundamental physics of F4GT rather than design realistic vdW heterostructures, we employ model leads in the monolayer device. This approach allows us to carry out efficient calculations, at a manageable computational cost, even at finite bias, while incorporating SOC or correlation effects within DFT+DMFT.

The model leads are effectively realized by considering a model metallic material with the same in-plane lattice parameters as F4GT and consisting of atoms with a single $6s$ orbital (with cut-off radius equal to about 6 Å). The atomic positions of the model leads are provided in Table S2. The model monolayer device is depicted in Fig. S2. In practical terms, the DOS of the F4GT monolayer with these leads remains very similar to that of a single F4GT layer within bulk F4GT, as demonstrated in Section S5 and illustrated in Fig. S16. Therefore, we are confident that conclusions drawn from the model monolayer device regarding, for example, the impact of correlations on the splitting of $3d$ orbitals, can be extrapolated

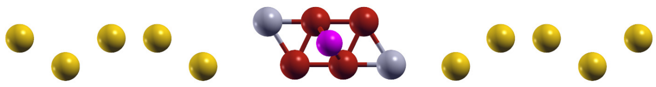

FIG. S2. Model monolayer device. Yellow, red, gray, and magenta spheres represent lead, Fe, Te, and Ge atoms, respectively.

to bulk or multilayered systems.

The distance between the leads and the F4GT layer (3.4 Å) is arbitrary. However, this mostly affects the broadening of the states, as we have verified. The predicted tunnelling current scales exponentially as a function of that distance. Hence the conductance's spin-polarization and the TMR ratio will remain constant as the spin-up and spin-down current decay in the same fashion.

atom 1	0.560	0.878	0.00
atom 2	2.267	0.878	2.414
atom 3	1.414	-0.600	4.828

TABLE S2. Atomic positions (in Å) of the model material used for the leads.

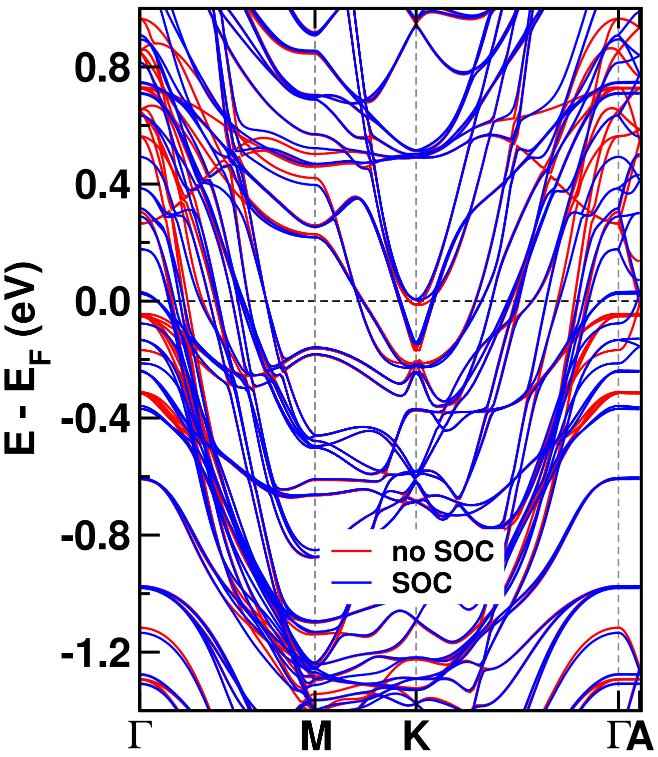

FIG. S3. Band structure of bulk F4GT with and without SOC.

S3. BAND STRUCTURE WITH SOC

Fig. S3 displays the band structure of bulk F4GT calculated with and without SOC (blue and red curves, respectively). The SOC has a quite strong effect around the Γ point, causing a large (∼ 100 meV) splitting of several bands, which would otherwise be degenerate. Moving along the Γ-A direction, which is relevant for perpendicular transport, we observe that there is only one dispersive band crossing the Fermi level, as explained in the paper. This band is weakly affected by the SOC, meaning that it preserves a distinct spin character, and therefore the transport is expected to remain half-metallic.

S4. COMPLEX BAND STRUCTURE OF BULK F4GT

The half-metallic character of bulk F4GT for perpendicular transport can be clearly visualized from the complex band structure[27–30], which is obtained as an output of the lead self-energies algorithm[16] implemented in Smeagol (an alternative and equally valid algorithm is described in Ref. [31]). The complex band structure generalizes the conventional band structure by considering wave-vectors with complex components and, therefore, describes the bulk-propagating states as well as the evanescent states that decay across the F4GT vdW gap.

Fig. S4 displays the calculated band structure (black lines) with real wave-vector k_z along the Γ-A direction in the BZ, and the complex band structure (blue lines) with $\mathrm{Im}[k_z] \neq 0$ at the Γ point and at the A point. The (a) and (b) panels are respectively for spin-up and down. The real band structure is identical to that presented in Fig. S3 and in Fig. 2 of the paper. For spin-up, there are only real bands, whereas, for spin-down, there is a band gap at the Fermi energy. The lowest spin-down conduction band terminates at the A point at an energy $E-E_\mathrm{F} \sim 0.15$ eV, and then continues as a complex band. Thus, while the transport of spin-up electrons is due to bulk-propagating states, the transport of spin-down electrons is due to evanescent states. As a consequence, the transmission obtained from the DFT-NEGF calculations differs by several order of magnitude for the two spin channels, resulting in the almost perfect spin-polarization of F4GT.

S5. DOS AND ORBITAL OCCUPATIONS

Fig. S5 displays the zero-bias spin-polarized DOS of the bulk and monolayer F4GT device, projected onto the two in-equivalent Fe atoms, Fe1 and Fe2.

In the bulk case, Fe1 has a stronger ferromagnetic character than Fe2. The spin-up 3d-PDOS at the Fermi level is larger for Fe2 than for Fe1. Furthermore, the two atoms also have a different spin-dependent filling. This

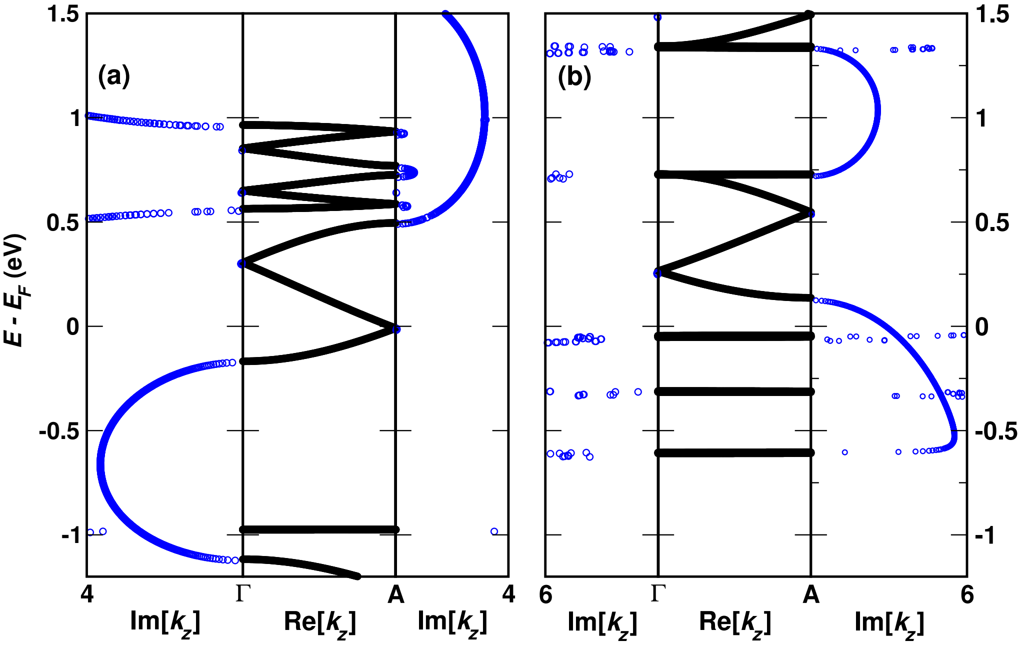

FIG. S4. Complex band structure of F4GT for (a) spin-up and (b) spin-down.

is seen from the Mulliken populations in Table S3. The average spin-up (spin-down) $3d$ orbital occupations are 0.92 (0.39) and 0.84 (0.48) electrons for Fe1 and Fe2, respectively. As such, the overall charge of Fe1 and Fe2 is the same, while their magnetic moments are about 2.7 μ_B and 1.8 μ_B.

In the monolayer case, we observe a reduction of the spin-splitting of the PDOS for both Fe atoms compared to the bulk case. Consequently, the magnetic moments from the Mulliken populations, which are reported in Table S4, are also slightly reduced. Nonetheless, we see that the magnetic moment of Fe1 still remains about 0.9 μ_B larger than the one of Fe2. This finding is consistent with the results of previous DFT calculations in the literature[32,33] (see also Section S8 E).

S6. PDOS ANALYSIS FOR THE F4GT MONOLAYER

Fe1, Fe2 and Te in an F4GT layer are aligned along the z transport direction in a chain-like fashion [see the inset of Fig. S5 and Fig. 1(b) in the paper]. Hence, the head-on overlap of their orbitals, forming σ covalent bonds, determine the effective delocalization of the electronic states, their coupling to the leads, and ultimately the transport properties. To see that, we present in Fig. S6(a), the zero-bias DOS projected over the $3d_{z^2}$ orbital of Fe1 (black curve) and Fe2 (red curve) and the $5p_z$ orbital of the Te atoms (blue curve), which, in the monolayer device, points towards the leads. We can then establish a clear correspondence between the PDOS and the zero-bias spin-dependent transmission coefficient, $T^\sigma(E)$, which is displayed Fig. 1(d) of the paper and which we show again in Fig. S6(b) in the interest of clarity.

In the spin-up channel, the Fe $3d_{z^2}$- and the Te $5p_z$-PDOS strongly overlap forming a very broad resonance

	$3d_{xy}$ ↑/↓	$3d_{yz}$ ↑/↓	$3d_{z^2}$ ↑/↓	$3d_{xz}$ ↑/↓	$3d_{x^2-y^2}$ ↑/↓	m (μ_B)
Fe1	0.93/0.33	0.88/0.39	0.96/0.5	0.88/0.39	0.93/0.33	2.72
Fe2	0.85/0.45	0.85/0.47	0.8/0.54	0.85/0.47	0.85/0.45	1.84

TABLE S3. Magnetic moment, m, and spin-up/down Mulliken populations of the Fe1 and Fe2 $3d$ orbitals for bulk F4GT.

	$3d_{xy}$ ↑/↓	$3d_{yz}$ ↑/↓	$3d_{z^2}$ ↑/↓	$3d_{xz}$ ↑/↓	$3d_{x^2-y^2}$ ↑/↓	m (μ_B)
Fe1	0.93/0.35	0.87/0.41	0.93/0.46	0.86/0.41	0.93/0.35	2.63
Fe2	0.85/0.47	0.85/0.48	0.81/0.5	0.84/0.49	0.84/0.47	1.75

TABLE S4. Magnetic moment, m, and spin-up/down Mulliken populations of the Fe1 and Fe2 $3d$ orbitals for the F4GT monolayer at zero bias.

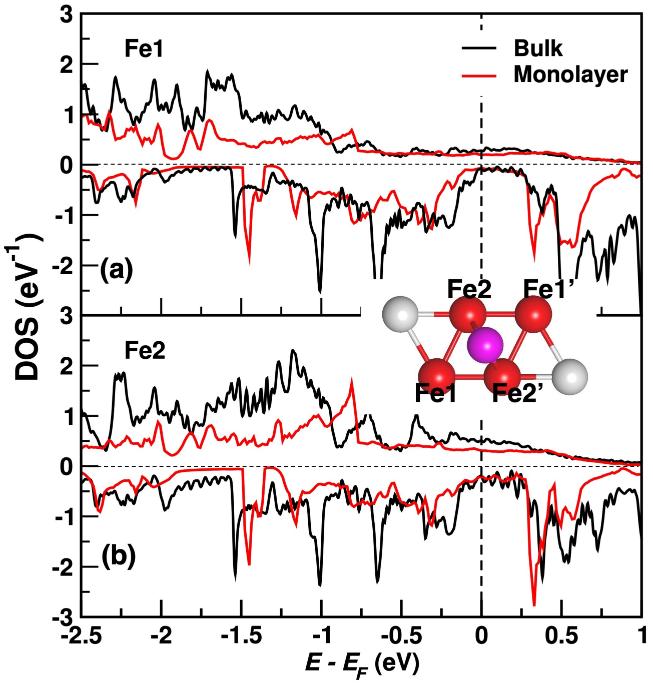

FIG. S5. Zero-bias DOS of bulk (black) and monolayer (red) F4GT projected over the Fe atoms. The panels (a) and (b) correspond to the two in-equivalent Fe atoms. The inset shows the F4GT monolayer where the in-equivalent Fe atoms are indicated. At zero-bias, Fe1 (Fe2) is equivalent to Fe1' (Fe2'). At finite-bias, all Fe atoms, Fe1, Fe2, Fe1', and Fe2', become in-equivalent. The gray and magenta spheres represent the Te and Ge atoms, respectively.

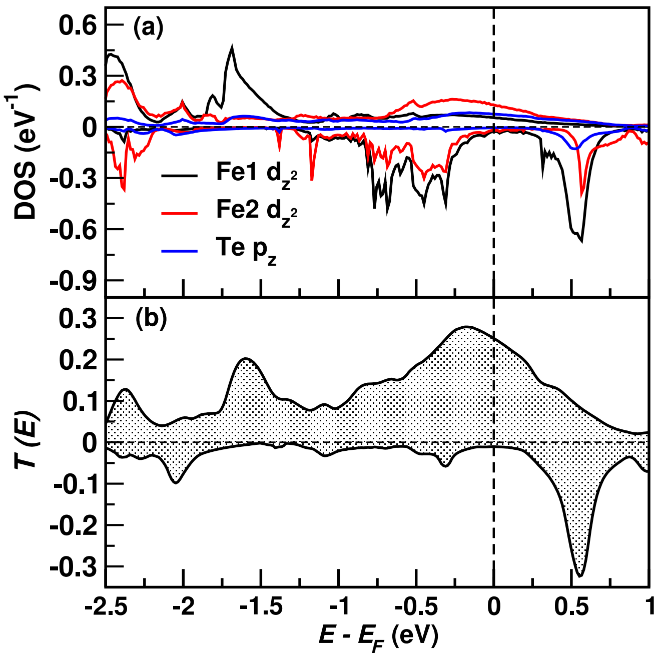

FIG. S6. (a) Zero-bias DOS projected over $3d_{z^2}$ orbitals of Fe1 (black), Fe2 (red) and Te (blue) $5p_z$. (b) Zero-bias transmission coefficient for the monolayer devices.

centered at $E - E_\mathrm{F} \approx -0.25$ eV. This means that the electronic states within that energy region extend across the whole F4GT layer's thickness. Furthermore, owing to the significant Te $5p_z$ orbital contribution, these states can also couple to the leads. This is the most ideal situation for transport as electrons incoming from one lead can be transmitted through the F4GT layer and toward the other lead with minimal scattering. Accordingly, the zero-bias spin-up transmission coefficient is found to have a pronounced peak with the same shape as the resonance in the PDOS at $E - E_\mathrm{F} \approx -0.25$ eV.

In the spin-down channel, the PDOS is gapped at E_F. The spin-down conduction states are located in energy at about $E - E_\mathrm{F} \approx 0.5$ eV, while the valence states span from about $E - E_\mathrm{F} \approx -0.25$ to -0.8 eV. Crucially, these valence and conduction states possess a different orbital decomposition.

As indicated by the PDOS, the spin-down conduction states have contributions from the Fe $3d_{z^2}$ as well as the Te $5p_z$ orbitals. As such, they are quite delocalized, can couple to the leads, and are expected to result in a large conductance. In fact, we observe that the spin-down transmission coefficient displays a sharp peak at $E - E_\mathrm{F} \approx 0.5$ eV, which is as high as the transmission resonance in the spin-up channel.

The spin-down valence states are mostly localized over the Fe core of the F4GT layer, as evident from the large Fe PDOS and the vanishing Te $5p_z$-PDOS between $E - E_\mathrm{F} \approx -0.25$ and -0.8 eV. Such localization translates in a low conductance. Accordingly, we see that the peak corresponding to the valence states at $E - E_\mathrm{F} \approx -0.3$ eV in the spin-down transmission coefficient is an order of magnitude smaller than the peak corresponding to the conduction states $E - E_\mathrm{F} \approx 0.5$ eV. Thus, there is a marked asymmetry in the spin-down transmission coefficient with respect to the center of the gap at E_F.

S7. ELECTRONIC STRUCTURE OF THE F4GT MONOLAYER AT FINITE-BIAS

The electronic structure of the F4GT monolayer considerably changes under an applied bias voltage. Specifically, the potential drop, $\Delta V_\mathrm{H}(z)$, across the device's central region, shown in Fig. S7, causes some intra-atomic charge redistribution, with all Fe atoms becoming in-equivalent as they are located at a different z coordinate along the device. This effect can be analyzed by comparing the Mulliken populations in Table S4 (zero-bias) with the ones in Table S5 (finite-bias, namely $V = 1$ V). The electron occupations of all atoms remain more or less constant. However, a fraction of the electron charge is transferred from the spin-up channel to the spin-down channel with V. Hence, there is a reduction of the Fe atoms' magnetic moments. This effect is particularly evident for Fe2 and Fe2'. At $V = 1$ V, their magnetic

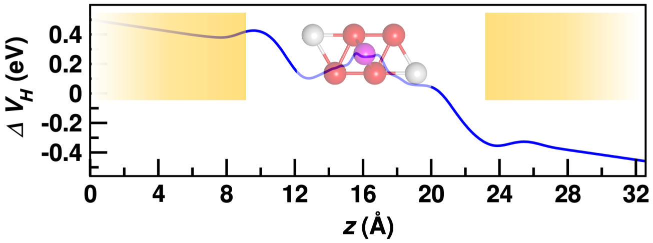

FIG. S7. Electrostatic potential drop $\Delta V_{\rm H}(z)$ across the central region of the monolayer device under a bias voltage, $V = 1$ V. The F4GT layer and the leads are represented in the background as a guide for the eyes. The red, gray, and magenta spheres represent the Fe, Te, and Ge atoms, respectively.

	$3d_{xy}$ ↑/↓	$3d_{yz}$ ↑/↓	$3d_{z^2}$ ↑/↓	$3d_{xz}$ ↑/↓	$3d_{x^2-y^2}$ ↑/↓	m ($\mu_{\rm B}$)
Fe1	0.91/0.37	0.86/0.42	0.89/0.46	0.86/0.43	0.91/0.37	2.48
Fe1'	0.91/0.37	0.87/0.4	0.91/0.45	0.87/0.4	0.92/0.37	2.62
Fe2	0.77/0.59	0.77/0.54	0.73/0.56	0.75/0.55	0.77/0.59	0.95
Fe2'	0.78/0.59	0.78/0.54	0.73/0.54	0.77/0.55	0.78/0.59	1.0

TABLE S5. Magnetic moment, m, and spin-up/down Mulliken populations of the $3d$ orbitals of all the Fe atoms in the the F4GT monolayer at $V = 1$ V.

moments become smaller than the corresponding zero-bias values by as much as $\sim 0.7\ \mu_B$. In practice, we find that the voltage bias modulates the magnetic properties of F4GT.

We may expect that the reduction of the Fe atoms' magnetic moments would be accompanied by the drastic drop of the spin-polarization at $E_{\rm F}$. However, that is not actually the case, as we see in the transmission coefficient in Fig. 1(d) of the paper. To understand this non-trivial finding, we compare the Fe atoms' d_{z^2}-PDOS at $V = 0$ V and $V = 1$ V in Fig. S8. In the spin-up channel, the bias induces a narrowing of the central conduction resonance, which is moreover shifted towards the Fermi energy, reflecting the reduction of the spin-up electron filling. This effect is compensated in the spin-down channel by an enhancement as well as a broadening of the valence resonance centered at $E - E_{\rm F} \approx -0.5$, accounting for the increasing spin-down occupation. In spite of that, the gap across $E_{\rm F}$, and therefore the half-metallic character of the system, remains unchanged.

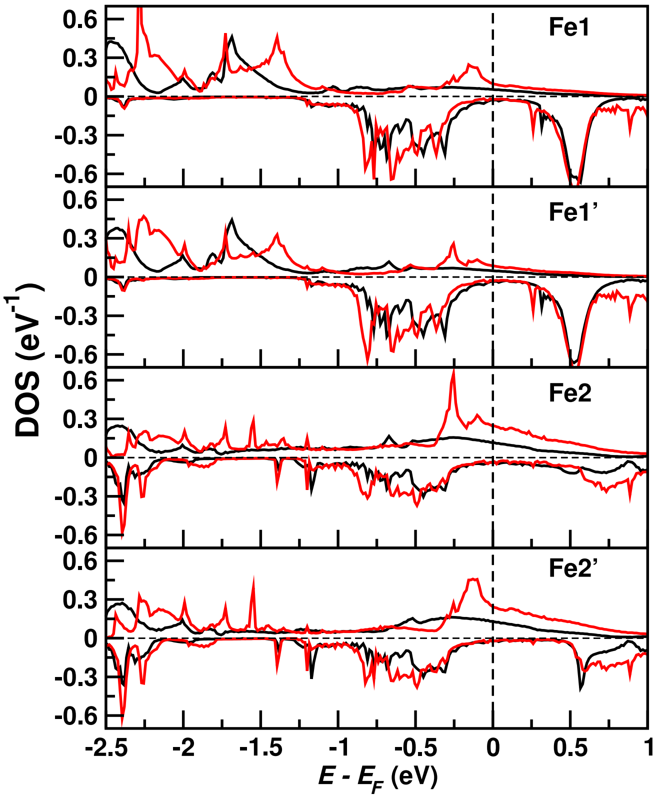

FIG. S8. d_{z^2}-PDOS of the monolayer Fe atoms at zero-bias (black) and at $V = 1$ V (red).

S8. ELECTRONIC STRUCTURE OF THE F4GT MONOLAYER PREDICTED BY DFT+U AND DFT+DMFT CALCULATIONS

A. DFT+U results

Figs. S9(a), (b), and (c) compare the zero-bias transmission coefficient and the Fe1 and Fe2 d_{z^2}-PDOS of the monolayer device calculated by DFT (black curve) and DFT+U (red curve) where $U = 3.0$ eV and $J = 0.5$ eV for all Fe atoms. The dependence of the results on the U and J parameters is discussed in Sec. S8 B.

In the spin-up channel, the Hubbard U-potential in Eq. (2) is negative as the Fe $3d$ orbitals have more than half-filled DFT occupations. Thus, DFT+U shifts the PDOS towards negative energies, leading to the complete occupation of Fe spin-up orbitals for both the in-equivalent Fe atoms. Consequently, we see that the broad resonance in the spin-up transmission function is translated in energy from $E_{\rm F}$ in DFT to $E - E_{\rm F} \sim -0.8$ eV in DFT+U.

In the spin-down channel, the U-potential is positive as the d orbitals are less than half-filled. Thus, the $3d$ orbitals move up in energy, further reducing their occupations. The peaks corresponding to the spin-down valence states are seen to cross the Fermi level, merging with the conduction states, so that the gap closes in the spin-down transmission coefficient, and the system loses its half-metallic character.

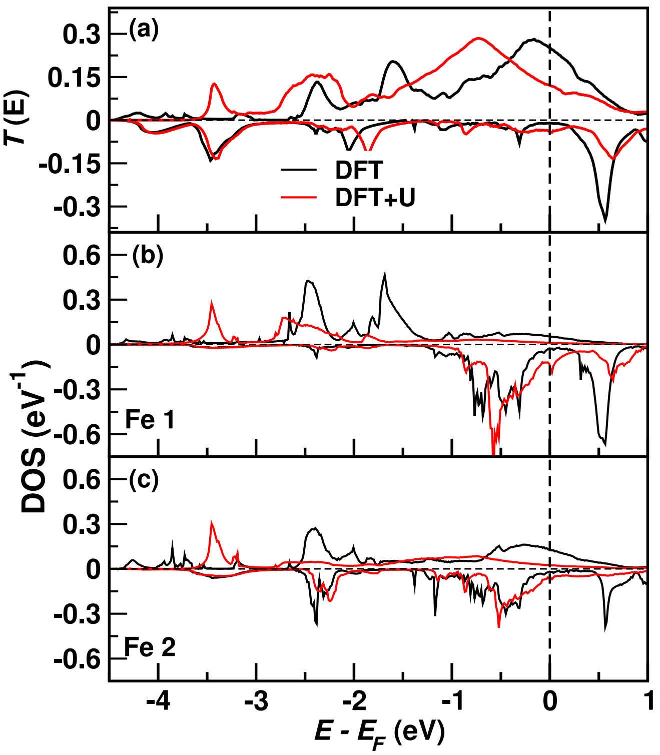

FIG. S9. DFT versus DFT+U results. (a) Transmission coefficient. (b) Fe1 d_{z^2}-PDOS. (c) Fe2 d_{z^2}-PDOS. The interaction parameters in DFT+U are $U = 3.0$ eV and $J = 0.5$ eV for both Fe atoms.

B. Dependence of the DFT+U results on the U interaction parameter

Fig. S10 compares the zero-bias transmission coefficient of the monolayer device calculated using DFT+U for $U = 2.5$ eV, $U = 3$ eV, and $U = 3.5$ eV (J is fixed at 0.5 eV). The most noticeable difference across the three cases is the shift of the resonance in the spin-up channel towards more negative energies, resulting from the increased potential on the $3d$ Fe orbitals as $(U - J)$ increases. Consequently, the spin-up transmission coefficient at E_F is progressively reduced. In contrast, the spin-down transmission coefficient is much less affected by the value of $(U - J)$, as the spin-down $3d$ orbitals are only a little less than half-filled, keeping the U-potential small.

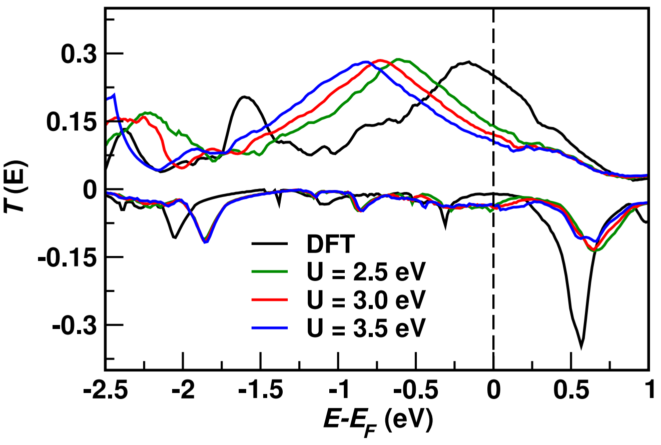

FIG. S10. Transmission coefficient calculated by using DFT+U with $U = 2.5$ eV, 3.0 eV, 3.5 eV, and $J = 0.5$ eV.

The systematic downward shift of the spin-up $3d$ orbitals with increasing $(U - J)$ is a common trend observed in all $3d$ ferromagnetic metals, and is due to an enhancement of the exchange splitting[15,19,34]. This shift correlates with the increase of the magnetic moments, as discussed below, in Section S8 E, but it leads to an unphysical description. Since DFT already overestimates the magnetic moments, and the U-potential further enhances this overestimation, DFT+U is not suitable for accurately describing the magnetism of F4GT[33] (and generally of any ferromagnetic metal). Dynamical correlation, as described in DMFT via an energy-dependent self-energy, becomes essential to compensate for the overestimation of the exchange splitting[35], and ultimately restores the half-metallic transmission.

C. DFT+DMFT results

The Fe1 and Fe2 d_{z^2}-PDOS calculated by DFT (black curve) and DFT+DMFT (red curve) are compared in Figs. S11(a) and (b). The real and imaginary parts of the DMFT self-energy of the Fe1 and Fe2 d_{z^2} orbitals are plotted in Fig. S11(c) and (d). Here, the U and J parameters used for the DFT+DMFT calculations are respectively equal to 3.0 eV and 0.5 eV. The dependence of the results on these parameters is analyzed in Sec. S8 D.

In the PDOS plot, we see that DMFT leads to a substantial redistribution of the spectral weight in both spin channels compared to DFT. This effect is due to the real part of the self-energy. Since $\mathrm{Re}[\Sigma^\sigma(E)]$ is positive and increases monotonically for $E < E_F$, the states below the Fermi level are drawn toward higher energies. In the spin-up channel, the PDOS resonance near the Fermi level gets narrow and sharp. In the spin-down channel, the center of valence states' resonance is translated up in energy by about 0.3 eV. Despite that, the resonance's edge remains pinned at the Fermi level without crossing it. The gap in the spin-down PDOS is not suppressed, although it is reduced.

The imaginary part of the DMFT self-energy, $\mathrm{Im}[\Sigma^\sigma(E)]$, is typical of a Fermi-liquid, i.e., $\mathrm{Im}[\Sigma^\sigma(E)] \propto (E - E_\mathrm{F})^2$ in both spin channels. Thus, no non-quasi-particle's features are found to emerge inside the gap in the spin-down channel. The F4GT monolayer in DFT+DMFT remains half-metallic like in DFT.

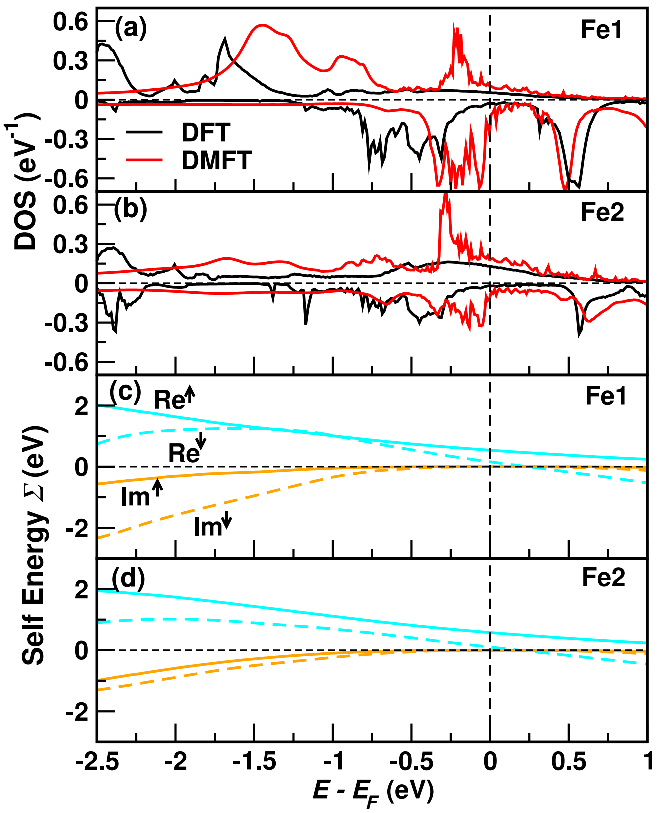

FIG. S11. d_{z^2}-PDOS for Fe1 (a) and Fe2 (b) calculated by DFT (black curve) and DFT+DMFT (red curve). Real (cyan) and imaginary (orange) part of the DMFT self-energy for the d_{z^2} orbitals of Fe1 (c) and Fe2 (d). The interaction parameters in DFT+DMFT are $U = 3.0$ eV and $J = 0.5$ eV for both Fe atoms.

D. Dependence of the DFT+DMFT results on the U interaction parameter

In Fig. S12, we show the zero-bias transmission coefficient of the monolayer device calculated by DFT+DMFT for several values of the local Coulomb interaction, U = 2.5 eV, $U = 3$ eV, and $U = 3.5$ eV (J is fixed at 0.5 eV). In all cases, the results look rather similar. In the spin-up channel, the main peak of the transmission coefficient is centered around the Fermi level, while, in the spin-down channel, there is a marked gap. The size of this gap tends to be reduced with U, but the effect is overall quite small. Therefore, the system is found to remain nearly half-metallic in all cases.

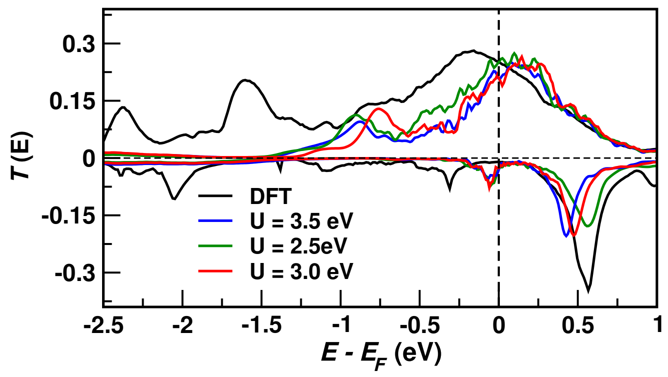

FIG. S12. Transmission coefficient calculated by using DFT and DFT+DMFT with $U = 2.5$ eV, 3.0 eV, 3.5 eV, and $J = 0.5$ eV.

E. Magnetic moments

Tab. S6 reports the magnetic moments calculated by using DFT, DFT+U, and DFT+DMFT for the in-equivalent Fe atoms at zero-bias. The DFT results agree remarkably well with those reported in literature[33], but overestimate the experimental values[25]. DFT+U further enhances this overestimation, with magnetic moments which increase with an increasing $(U - J)$ value. Hence, the method does not accurately capture magnetism in this system. This trend is consistent with the one reported in Ref. [33]. For all values of U considered within DFT+DMFT, we find that the Fe2 moment is enhanced while the Fe1 moment is reduced with respect to DFT. This contradicts the findings of Ref. [33], where they observed the opposite trend in their DFT+DMFT calculations: a reduction in the Fe2 moment and enhancement in the Fe1 moment. The origin of this discrepancy may be due to our calculations using the same value of U on both in-equivalent Fe atoms, while Ref. [33] applies an atom specific $U_{\mathrm{Fe1}} > U_{\mathrm{Fe2}}$, obtained from constrained linear-response calculations. This issue is however not particularly important for transport as suggested by the poor dependence of the transmission coefficient on U (see Section S8 D and Fig. S12).

	Fe1	Fe2	Average
DFT	2.63	1.75	2.19
DFT+U ($U = 2.5$ eV)	2.90	2.22	2.56
DFT+U ($U = 3.0$ eV)	2.95	2.29	2.62
DFT+U ($U = 3.5$ eV)	3.02	2.38	2.70
DFT+DMFT ($U = 2.5$ eV)	2.58	2.02	2.30
DFT+DMFT ($U = 3.0$ eV)	2.54	2.00	2.27
DFT+DMFT ($U = 3.5$ eV)	2.45	1.87	2.16

TABLE S6. Magnetic moments (in μ_B) of the two in-equivalent Fe atoms in the F4GT monolayer at zero-bias for all calculations.

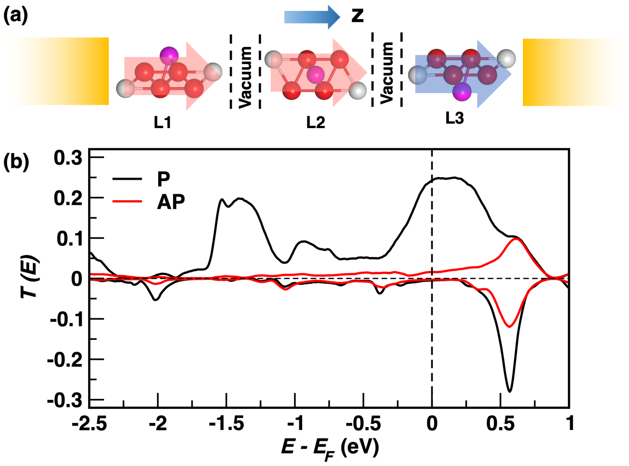

FIG. S13. Transport calculations for the trilayer MTJ device. (a) Schematic representation of the device. The three F4GT layers are labelled L1, L2 and L3, and are sandwiched between model leads (semi-infinite yellow rectangles). (b) Zero-bias transmission coefficient for the P and AP configurations.

S9. RESULTS FOR A MTJ COMPRISING THREE F4GT LAYERS

We present here the results for the device shown in Fig. S13(a) where the central region consists of three F4GT layers separated by a vdW gap. The first two layers, denoted as L1 and L2, serve as spin-filter, while the third layer, L3, functions as a spin detector. The central region is connected to the same model leads as in the monolayer and bilayer devices. We note that the atoms in the central region are assumed to be at the same positions as in the bulk primitive unit cell (i.e., the atomic coordinates are the same as in Table S1), and no geometry relaxation is carried out. This is adequate for the scope of this section, which is to provide some indications on general trends.

The device is set in two different magnetic configurations. In the first configuration, the magnetization vectors of three layers are parallel (P) to each other, while in the second, the magnetization vectors of L1 and L2 are antiparallel (AP) to the magnetization vector of L3. The zero-bias P and AP transmission coefficients for both spin channels, $T^{\sigma}_{\mathrm{P}}(E)$ and $T^{\sigma}_{\mathrm{AP}}(E)$, are shown in Fig. S13(b). In the P configuration, the transmission coefficient closely resembles that of the monolayer and bilayer devices, exhibiting a half-metallic character. There is a broad resonance around E_{F} in the spin-up channel and a gap in the spin-down channel. On the other hand, in the AP configuration, the transmission coefficient is dramatically reduced in both spin channels. Furthermore, $T^{\uparrow}_{\mathrm{AP}}(E)$ and $T^{\downarrow}_{\mathrm{AP}}(E)$ are not identical. This is because the AP configuration is asymmetric, as the magnetization vectors of the first two layers are aligned opposite to the magnetization vector of the third layer. In mathematical terms, this asymmetry becomes evident by expressing the spin-up and down AP transmission coefficients as square root of the products of the spin-up and down transmission coefficients in the P case, namely $T^{\uparrow}_{\mathrm{AP}}(E) \approx \sqrt{T^{\uparrow}_{\mathrm{P}}(E)T^{\uparrow}_{\mathrm{P}}(E)T^{\downarrow}_{\mathrm{P}}(E)}$ and $T^{\downarrow}_{\mathrm{AP}}(E) \approx \sqrt{T^{\downarrow}_{\mathrm{P}}(E)T^{\downarrow}_{\mathrm{P}}(E)T^{\uparrow}_{\mathrm{P}}(E)}$, according to a standard model of MTJs[14,36].

Finally, the TMR ratio at zero-bias, defined as

$$\mathrm{TMR} = \frac{T_{\mathrm{P}}(E_{\mathrm{F}}) - T_{\mathrm{AP}}(E_{\mathrm{F}})}{T_{\mathrm{AP}}(E_{\mathrm{F}})} \tag{3}$$

with $T_{\mathrm{P(AP)}}(E) = T^{\uparrow}_{\mathrm{P(AP)}}(E) + T^{\downarrow}_{\mathrm{P(AP)}}(E)$, is calculated to reach an impressive 1200%. Such a high predicted value is comparable to the one obtained for Fe(001)/MgO MTJs[37] which is employed in technological applications.

S10. FERMI SURFACES AND ZERO-BIAS k-RESOLVED TRANSMISSION COEFFICIENTS

A. Bulk

The paper explains the spin-polarized linear-response transport through F4GT by examining its band structure. It specifically addresses the presence or absence of spin-up and spin-down bands at E_{F} along the Γ-A direction, which corresponds to states with the transverse wave-vector $\mathbf{k}_{\parallel} = 0$ in the 2D BZ used in DFT-NEGF calculations. However, it is important to note that, in addition to these states, there may be others with transverse wave-vector $\mathbf{k}_{\parallel} \neq 0$ that were neglected in the simplified band structure analysis but could contribute to the zero-bias conductance. To reveal these additional states, we

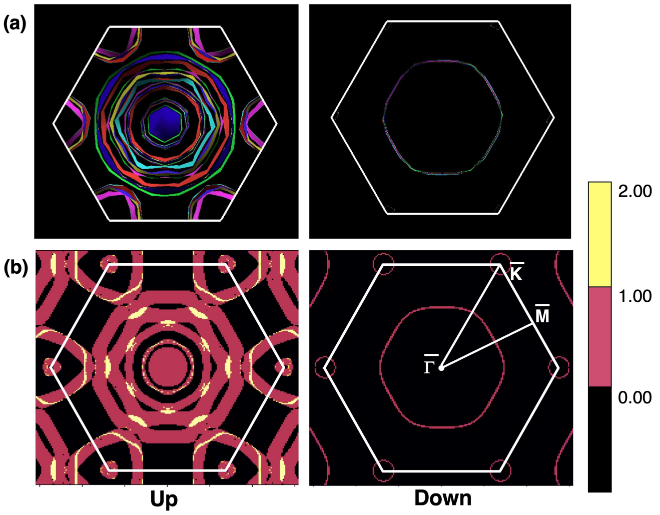

FIG. S14. (a) Spin-dependent Fermi surface of bulk F4GT (different colours correspond to different bands). (b) Spin- and $\mathbf{k}_{\parallel}$-resolved transmission coefficient of bulk F4GT at E_{F}. The white hexagon denotes the boundaries of the 2D BZ.

present the spin-dependent Fermi surface projected onto the transport direction in Fig. S14(a).

The spin-up Fermi surface [left panel in Fig. S14(a)] consists of numerous Fermi sheets covering a large portion of the 2D BZ. This means that there are many bands with different $\mathbf{k}_{\|}$ that cross the Fermi level (different colours correspond to different bands). The spin-up Fermi surface displays a prominent circle at the $\bar{\Gamma}$ point, followed by concentric Fermi sheets forming polygonal rings with an increasing number of sides as their radii increase. Similar polygonal rings are also present around the $\bar{\mathrm{K}}$ points. In contrast, the spin-down Fermi surface [right panel in Fig. S14(a)] features primarily an isolated sheet, appearing as a hexagonal ring around the $\bar{\Gamma}$ point, which is accompanied by additional rings around the $\bar{\mathrm{K}}$ points.

At the quantitative level, in DFT-NEGF calculations, the number of conduction states (also called "channels") for each wave-vector $\mathbf{k}_{\|}$ is obtained from the zero-bias transmission coefficient in the 2D BZ at the Fermi energy, $T^{\sigma}(E_{\mathrm{F}}, \mathbf{k}_{\|})$ as plotted in Fig. S14(b). Since, in bulk F4GT, the leads and the central region are made of the same material, there is no scattering in the system. Thus, the transport is ballistic, and $T^{\sigma}(E_{\mathrm{F}}, \mathbf{k}_{\|})$ is an integer, specifically in this case either 0, 1 or 2, for each transverse wave-vector $\mathbf{k}_{\|}$. The distribution of the spin-up and spin-down conduction channels mirrors the corresponding Fermi surface, revealing that there are few conducting spin-down channels alongside numerous spin-up channels. Although the number of spin-down channels is negligible compared to the number of spin-up channels, the presence of those few spin-down channels slightly reduces the spin-polarization from 1 to 0.92.

Notably, while $T^{\sigma}(E_{\mathrm{F}}, \mathbf{k}_{\|})$ is an integer for each $\mathbf{k}_{\|}$, the spin-up and spin-down transmission coefficient, $T^{\sigma}(E)$, depicted in Fig. 1(c) of the paper, is not because $T^{\sigma}(E)$ is summed over all $\mathbf{k}_{\|}$ in the 2D BZ, according to Eq. (1).

The Fermi surface of bulk F4GT in Fig. S14(b) can be directly compared to the Fermi surface of the related compound Fe_3GeTe_2 (F3GT), displayed in Fig. 2(a) and (b) of Ref. [38] by Li *et al.*. The spin-up Fermi surfaces of both materials cover a large portion of the 2D BZ. Despite their general similarity, several differences are notable. Firstly, F3GT exhibits an extended Fermi sheet along the $\bar{\Gamma}$-$\bar{\mathrm{M}}$ direction, which is instead absent in F4GT. Secondly, F3GT features a Fermi sheet covering each $\bar{\mathrm{K}}$ point, whereas F4GT displays a series of concentric polygonal rings around $\bar{\mathrm{K}}$. Lastly, the radius of the circle covering $\bar{\Gamma}$ is smaller for F3GT than for F4GT. Thus, in F4GT, the main contribution to spin-up transport originates primarily from channels near $\bar{\Gamma}$, unlike in F3GT, where there are also channels around $\bar{\mathrm{M}}$ and $\bar{\mathrm{K}}$.

The differences between F4GT and F3GT are more pronounced in their spin-down Fermi surface. Although both materials display an isolated hexagonal sheet around $\bar{\Gamma}$, F3GT features additional circles covering $\bar{\Gamma}$ and around $\bar{\mathrm{K}}$, which are less prominent or absent

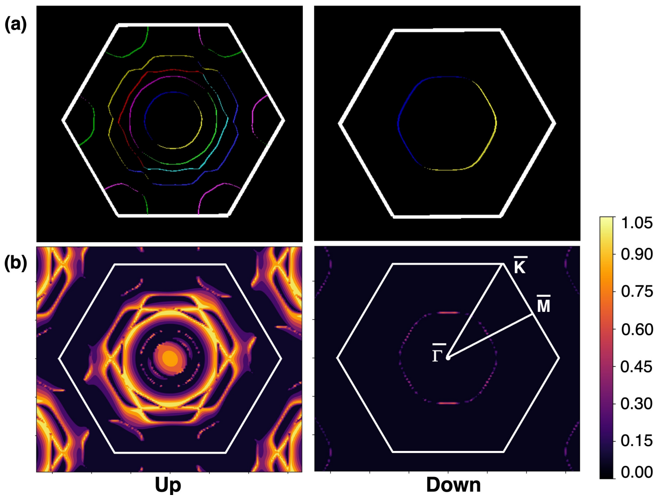

FIG. S15. (a) Spin-dependent Fermi surface of the F4GT monolayer (different colours corresponds to different bands). (b) Spin- and $\mathbf{k}_{\|}$-resolved transmission coefficient of F4GT monolayer at E_{F}. The white hexagon denotes the boundaries of the 2D BZ.

in F4GT. As a result, F3GT possesses more spin-down transport channels compared to F4GT, leading to a lower spin-polarization conductance than that of F4GT.

Interestingly, the Fermi surface of F4GT resembles the constant energy surface of F3GT at an energy $E_{\mathrm{F}} - 0.3$ eV, as reported in Fig. 6 of Ref. [38]. This similarity suggests that the spin transport properties of F4GT could potentially be mimicked by p-doping F3GT, assuming doping induces a rigid band shift. In this hypothetical scenario, Ref. [38] predicts nearly half-metallic conduction in F3GT, akin to our findings for F4GT in this study.

B. Monolayer

The study of the spin-dependent Fermi surface and of the $\mathbf{k}_{\|}$-resolved transmission coefficient, $T^{\sigma}(E_{\mathrm{F}}, \mathbf{k}_{\|})$, can be extended from bulk F4GT to the monolayer.

The Fermi surface of the isolated monolayer (i.e., the monolayer not attached to the leads) is depicted in Fig. S15(a). It is qualitatively similar to that of the bulk F4GT. For spin-up electrons, we observe several concentric Fermi sheets centered at $\bar{\Gamma}$, forming polygonal rings with an increasing number of sides as their radii increase. These are accompanied by isolated rings around the $\bar{\mathrm{K}}$ points. The primary difference with the spin-up Fermi surface of bulk F4GT is the absence of a circle covering the $\bar{\Gamma}$ point.

For spin-down electrons, the Fermi surface of the monolayer is almost identical to that of bulk F4GT. It primarily consists of a single localized hexagonal ring around the $\bar{\Gamma}$ point.

The zero-bias $\mathbf{k}_{\|}$-resolved transmission coefficient at the Fermi energy, $T^{\sigma}(E_{\mathrm{F}}, \mathbf{k}_{\|})$, of the monolayer device is shown in Fig. S15(b). Unlike the bulk F4GT case,

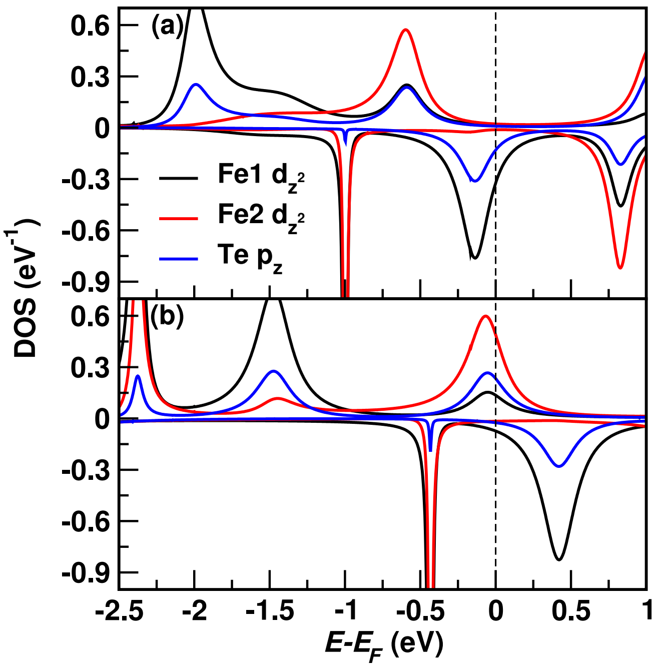

FIG. S16. Zero-bias PDOS for $\mathbf{k}_{\|} = (0, 0)$ Å^{-1} of the isolated F4GT monolayer (a) and the F4GT monolayed contacted to the model leads (b). The PDOS of the isolated monolayer is arbitrarily broadened to resemble the PDOS of the contacted monolayer, where the broadening is instead caused by the electronic coupling to the leads.

for the monolayer device $T^\sigma(E_\mathrm{F}, \mathbf{k}_\|)$ does not assume integer values due to elastic scattering of electrons at the interfaces between the model leads and the F4GT layer. However, it generally resembles the previous result for bulk F4GT. There are many highly conducting spin-up channels with $T^\sigma(E_\mathrm{F}, \mathbf{k}_\|) > 0.7$, while there are only a few spin-down conducting channels with $T^\sigma(E_\mathrm{F}, \mathbf{k}_\|)$ equal to just about 0.3. As such, the F4GT monolayer device displays nearly half-metallic conductance, similar to the one of the bulk system.

$T^\sigma(E_\mathrm{F}, \mathbf{k}_\|)$ mirrors the corresponding Fermi surface of the isolated monolayer, except for the notable presence of spin-up transport channels at $\bar{\Gamma}$ in the 2D BZ, where the Fermi surface instead displays no Fermi sheets at all. This difference is due to the modification of the electronic structure of the F4GT layer induced by the hybridization with the s states of the leads. To see that, we display the Fe d_{z^2}- and Te p_z-PDOS of both the isolated monolayer and the monolayer between the leads for $\mathbf{k}_\| = (0, 0)$ Å^{-1} in Fig. S16. In the isolated monolayer [Fig. S16(a)], the spin-up PDOS vanishes at E_F, consistent with the Fermi surface plot showing no spin-up states at $\bar{\Gamma}$ in the 2D BZ. The first spin-up resonance in the PDOS is observed at $E - E_\mathrm{F} \approx -0.6$ eV. After contacting the layer with the leads [Fig. S16(b)], this resonance shifts up in energy, eventually reaching E_F and giving rise to the open transport channel at $\bar{\Gamma}$.

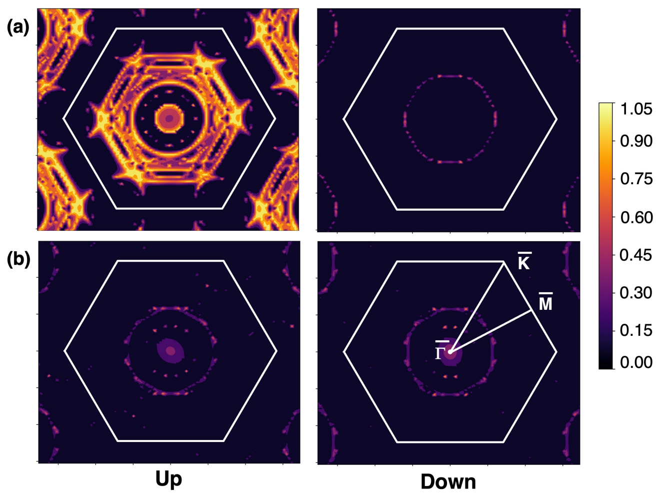

FIG. S17. Spin- and $\mathbf{k}_\|$-resolved transmission coefficient of the F4GT-based MTJ at E_F for the P (a) and AP (b) configurations. The white hexagon denotes the boundaries of the 2D BZ.

C. F4GT-based MTJ

The spin-dependent $\mathbf{k}_\|$-resolved transmission coefficient at the Fermi level, $T^\sigma_{\mathrm{P(AP)}}(E_\mathrm{F}, \mathbf{k}_\|)$, of the F4GT-based MTJ device is shown in Figs. S17(a) and (b) for the P and AP configurations, respectively.

Since the wave-vector and spin are conserved in the electron tunnelling process, the difference between $T^\sigma_\mathrm{P}(E_\mathrm{F}, \mathbf{k}_\|)$ and $T^\sigma_\mathrm{AP}(E_\mathrm{F}, \mathbf{k}_\|)$ can intuitively be understood in terms of the transmission coefficient of each individual layer in the junction, $T^\sigma_1(E_\mathrm{F}, \mathbf{k}_\|)$ and $T^\sigma_2(E_\mathrm{F}, \mathbf{k}_\|)$, which can be both further approximated by the transmission coefficient of the monolayer device from the previous section [namely, Fig. S15(b)].

In the P configuration, the transmission coefficient of the first and second layer overlap exactly for both spin channels, allowing states at the Fermi level with a wave-vector $\mathbf{k}_\|$ incoming from one layer to be transmitted through the other layer. In first approximation and neglecting quantum interference effects, $T^\uparrow_\mathrm{P}$ and $T^\downarrow_\mathrm{P}$ are respectively equal to $T^\uparrow_1 T^\uparrow_2$ and $T^\downarrow_1 T^\downarrow_2$. In contrast, in the AP configuration, the spin-down and spin-up states of the second layer are swapped relative to those of the first layer. Hence, $T^\uparrow_\mathrm{AP}$ and $T^\downarrow_\mathrm{AP}$ are respectively equal to $T^\uparrow_1 T^\downarrow_2$ and $T^\downarrow_1 T^\uparrow_2$. Since $T^\uparrow_{1(2)} >> T^\downarrow_{1(2)} \approx 0$ over a large area of the 2D BZ, the zero-bias conductance of the P configuration will be much larger than that of the AP configuration, resulting in a large TMR. We note that, due to the AB stacking of two F4GT layers, our system lacks inversion symmetry, resulting in the AP spin-up and spin-down $\mathbf{k}_\|$-resolved transmission coefficients in Fig. S15(b) not being identical.

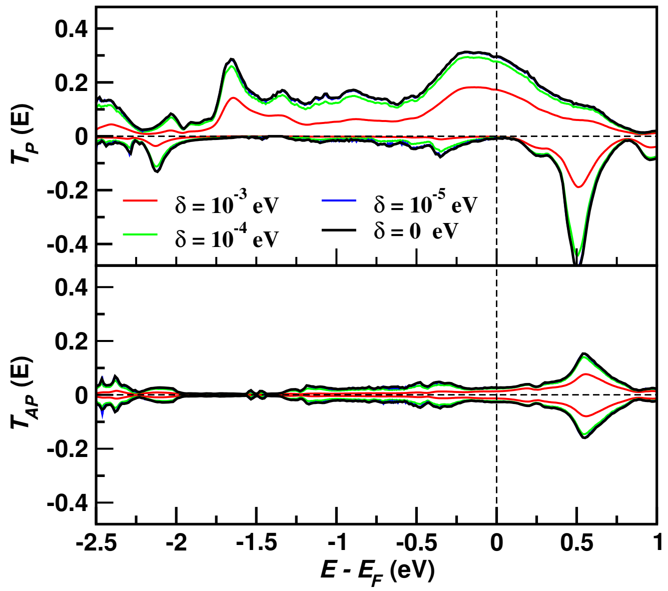

FIG. S18. Zero-bias transmission coefficient of the bilayer MTJ for different δ values. The top panel is for the P configuration, while the bottom panel is for the AP configuration.

S11. DISORDER, STRAIN, AND WORK FUNCTION EFFECTS

In this section, we present several test calculations to gain qualitative insights into how disorder, strain, and doping, which are likely to be present in real experimental devices, could potentially degrade the nearly perfect spin transport properties of our model F4GT monolayer and bilayer junctions.

A. TMR of the F4GT MTJ with disorder

The states responsible for the spin-polarized conduction in F4GT predominately arise from the Fe $3d_{z^2}$ orbitals which may be very susceptible to scattering due to disorder. Disorder is commonly neglected in first-principles studies as the accurate description would require large supercells and averaging over numerous possible random disorder realizations, making the calculations too computationally demanding. Nonetheless, the effect of disorder within DFT+NEGF can be approximately modeled by adding an imaginary part, denoted as δ, to the energy when computing the retarded Green's function[39]. This approach introduces a uniform broadening of conducting states and represents unstructured disorder. Despite its simplicity, the approximation can already provide some qualitative indications of disorder effects on the TMR, as demonstrated in the past for prototypical Fe/MgO/Fe MTJs[40,41].

The zero-bias spin-dependent transmission coefficient of the bilayer MTJ for four values of δ is shown in Fig. S18 for both the parallel P and AP configurations. We can see that increasing δ leads to a gradual reduction of both $T_{\rm P}(E)$ and $T_{\rm AP}(E)$. The corresponding changes in the zero-bias TMR ratio are reported in Table S7. Overall, we observe a systematic enhancement of the TMR with disorder, going from about 450% for $\delta = 0$ eV (the value currently reported in the manuscript) to 560% for $\delta = 10^{-3}$ eV. In summary, we find that the system's spin-transport performances remain quite robust against disorder within the assumed approximation.

δ (eV)	0	10^{-5}	10^{-4}	10^{-3}
TMR	456%	477%	503%	557%

TABLE S7. TMR ratio as a function of δ.

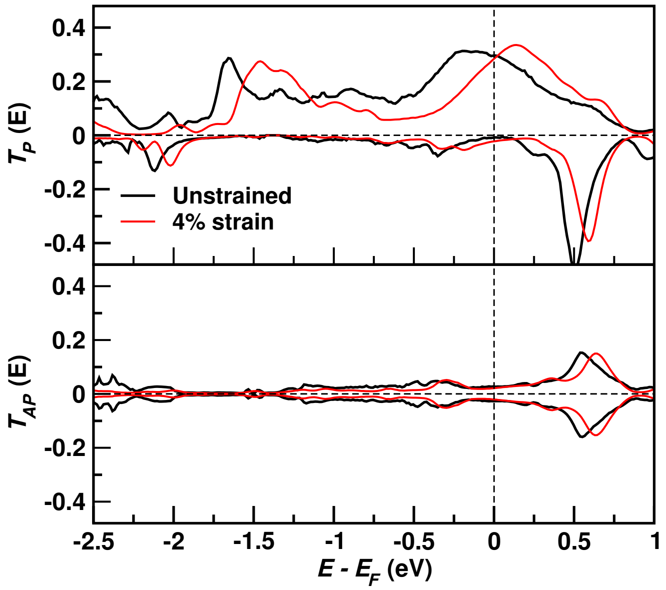

FIG. S19. Zero-bias P and AP transmission coefficients of the F4GT bilayer MTJ with 4% strain (red) and without strain (black).

B. Spin-polarization of a strained F4GT bilayer

Real MTJs used in experiments typically consist of multiple layers of different materials, requiring matching different structures and lattice parameters. This means that the magnetic layers will experience strain and potential distortion. Although a detailed study of these effects is beyond the scope of this work, we consider here a bilayer F4GT system subjected to a significant in-plane 4% tensile strain as an extreme test case to investigate whether the zero-bias spin transport properties of F4GT are affected. Such strain is practically applied by changing the lattice parameter, a, from 3.93 Å to 4.077 Å.

The zero-bias transmission coefficients for the P and AP configurations of the strained and unstrained bilayer

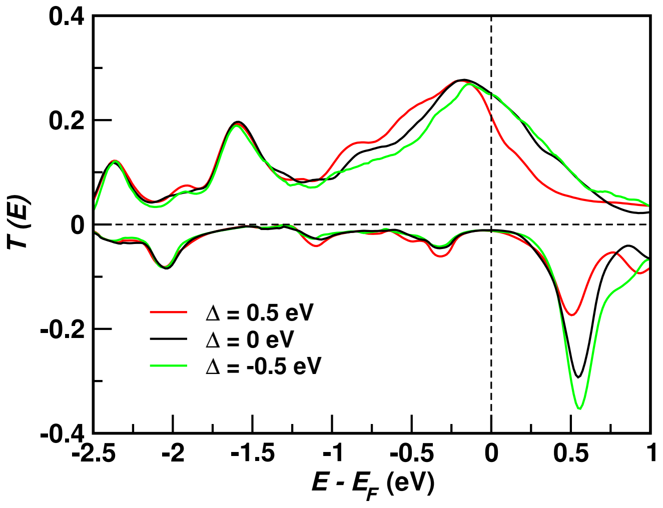

FIG. S20. Comparison of zero-bias transmission coefficients of the monolayer device with the leads' work function changed by $\Delta = 0.5$ eV (red curve) and -0.5 eV (green curve) with respect to its original value ($\Delta = 0$ eV -black curve). Note that, although the calculations are practically performed by varying E_{F} and keeping E_{v} constant, the results are here presented with E_{F} aligned at 0 eV (and, therefore, shifting E_{v} by Δ) in all cases, for a better comparison.

MTJs are compared in Fig. S19. In both configurations, we observe an almost rigid shift of the transmission coefficient of the strained system towards high energies compared to the unstrained one. In the P configuration, this causes the lower edge of the band gap in the spin-down channel to move closer to E_{F}. As a result the spin-polarization is slightly reduced. However, the effect is quantitatively small. In the AP configuration, the modification of the transmission coefficient is negligible over an approximately 0.5 eV-wide energy region around E_{F}, leaving the transport properties unchanged. In summary, the TMR will be nearly unaffected by the applied strain, further indicating the robustness of the system and its potential for applications in real devices.

C. Effect of the leads' work function on the spin-polarization of the F4GT monolayer

In the study of the F4GT devices, the use of model leads allows us to understand the basic physics of the system. However, in real junctions, different work functions of the metal electrodes may induce doping of the F4GT layers, potentially altering the transmission coefficient. Here, we briefly assess whether this effect might degrade the predicted nearly perfect spin-polarization in the case of the monolayer device.

The work function of metallic leads is defined as the difference between the vacuum energy and the Fermi energy, WF $= E_{\mathrm{v}} - E_{\mathrm{F}}$. In our calculations, E_{v} is estimated by considering a device without any F4GT layers, placing the leads at a large distance one from the other, and taking the value of the Hartree potential at the center of the vacuum region in between the leads. As a result, for our model system, we obtain WF ~ 4.2 eV, which is a general realistic value for metals. The effect of changing the leads' work function in the monolayer device can then be simulated by varying the value of E_{F} while E_{v} remains constant. Specifically, here, we shift E_{F} by $\Delta = \pm 0.5$ eV (which corresponds to changing WF by $-\Delta$) and, for each case, re-perform the self-consistent calculations for the central region. The obtained zero-bias transmission coefficients are displayed Fig. S20.

The zero-bias transmission coefficient displays some quantitative changes when varying WF. Specifically, in the spin-up channel, it is slightly reduced at E_{F} for $\Delta = 0.5$ eV compared to the other cases. On the other hand, in the spin-down channel, we observe that the height of the resonance at $E - E_{\mathrm{F}} \approx 0.5$ eV increases with increasing the work function. Despite that, the transport gap in the spin-down channel is preserved and appears nearly identical in all three presented cases. As a consequence, the conductance spin-polarization is barely modified, being 0.91, 0.92, and 0.89 for $\Delta = -0.5$, 0 and $\Delta = 0.5$ eV, respectively. In other words, the zero-bias conductance remains nearly half-metallic, regardless of the leads' work function.

* andrea.droghetti@tcd.ie

[1] Soler, J. M.; Artacho, E.; Gale, J. D.; García, A.; Junquera, J.; Ordejón, P.; Sánchez-Portal, D. The SIESTA method forab initioorder-Nmaterials simulation. *Journal of Physics: Condensed Matter* **2002**, *14*, 2745–2779.

[2] García, A. et al. Siesta: Recent developments and applications. *The Journal of Chemical Physics* **2020**, *152*, 204108.

[3] Perdew, J. P.; Burke, K.; Ernzerhof, M. Generalized Gradient Approximation Made Simple. *Phys. Rev. Lett.* **1996**, *77*, 3865–3868.

[4] Grimme, S.; Antony, J.; Ehrlich, S.; Krieg, H. A consistent and accurate ab initio parametrization of density functional dispersion correction (DFT-D) for the 94 elements H-Pu. *The Journal of Chemical Physics* **2010**, *132*, 154104.

[5] Troullier, N.; Martins, J. L. Efficient pseudopotentials for plane-wave calculations. *Phys. Rev. B* **1991**, *43*, 1993–2006.

[6] Troullier, N.; Martins, J. L. Efficient pseudopotentials for plane-wave calculations. II. Operators for fast iterative diagonalization. *Phys. Rev. B* **1991**, *43*, 8861–8869.

[7] Artacho, E.; Sánchez-Portal, D.; Ordejón, P.; García, A.; Soler, J. M. Linear-Scaling ab-initio Calculations for Large and Complex Systems. *physica status solidi (b)* **1999**, *215*, 809–817.

[8] Junquera, J.; Paz, Ó.; Sánchez-Portal, D.; Artacho, E. Numerical atomic orbitals for linear-scaling calculations. *Physical Review B* **2001**, *64*, 235111.

[9] Rivero, P.; García-Suárez, V. M.; Pereñiguez, D.; Utt, K.; Yang, Y.; Bellaiche, L.; Park, K.; Ferrer, J.; Barraza-Lopez, S. Systematic pseudopotentials from reference eigenvalue sets for DFT calculations. *Computational Materials Science* **2015**, *98*, 372–389.

[10] Kokalj, A. XCrySDen—a new program for displaying crystalline structures and electron densities. *Journal of Molecular Graphics and Modelling* **1999**, *17*, 176–179.

[11] Fernández-Seivane, L.; Oliveira, M. A.; Sanvito, S.; Ferrer, J. On-site approximation for spin–orbit coupling in linear combination of atomic orbitals density functional methods. *Journal of Physics: Condensed Matter* **2006**, *18*, 7999.

[12] Kresse, G.; Hafner, J. Ab initio molecular dynamics for liquid metals. *Phys. Rev. B* **1993**, *47*, 558–561.

[13] Rocha, A. R.; García-Suárez, V. M.; Bailey, S.; Lambert, C.; Ferrer, J.; Sanvito, S. Spin and molecular electronics in atomically generated orbital landscapes. *Phys. Rev. B* **2006**, *73*, 085414.

[14] Rungger, I.; Droghetti, A.; Stamenova, M. In *Handbook of Materials Modeling. Vol. 1 Methods: Theory and Modeling*; Yip, S., W. Andreoni, W., Eds.; Springer International Publishing, 2019.

[15] Droghetti, A.; Radonjić, M. M.; Chioncel, L.; Rungger, I. Dynamical mean-field theory for spin-dependent electron transport in spin-valve devices. *Phys. Rev. B* **2022**, *106*, 075156.

[16] Rungger, I.; Sanvito, S. Algorithm for the construction of self-energies for electronic transport calculations based on singularity elimination and singular value decomposition. *Phys. Rev. B* **2008**, *78*, 035407.

[17] Droghetti, A.; Rungger, I.; Rubio, A.; Tokatly, I. V. Spin-orbit induced equilibrium spin currents in materials. *Phys. Rev. B* **2022**, *105*, 024409.

[18] Bajaj, A.; Gupta, R.; Tokatly, I. V.; Sanvito, S.; Droghetti, A. Ab initio transport theory for the intrinsic spin Hall effect applied to 5d metals. *Phys. Rev. B* **2024**, *109*, 195132.

[19] Droghetti, A.; Radonjić, M. M.; Halder, A.; Rungger, I.; Chioncel, L. DFT + Σ_2 method for electron correlation effects at transition metal surfaces. *Phys. Rev. B* **2022**, *105*, 115129.

[20] Droghetti, A.; Rungger, I. Quantum transport simulation scheme including strong correlations and its application to organic radicals adsorbed on gold. *Phys. Rev. B* **2017**, *95*, 085131.

[21] Imada, M.; Fujimori, A.; Tokura, Y. Metal-insulator transitions and Correlated Metals in d-Electron Systems. *Rev. Mod. Phys.* **1998**, *70*, 1039–1263.

[22] Anisimov, V. I.; Gunnarsson, O. Density-functional calculation of effective Coulomb interactions in metals. *Phys. Rev. B* **1991**, *43*, 7570–7574.

[23] Dudarev, S. L.; Botton, G. A.; Savrasov, S. Y.; Humphreys, C. J.; Sutton, A. P. Electron-energy-loss spectra and the structural stability of nickel oxide: An LSDA+U study. *Phys. Rev. B* **1998**, *57*, 1505–1509.

[24] Su, Y.; Li, X.; Zhu, M.; Zhang, J.; You, L.; Tsymbal, E. Y. Van der Waals Multiferroic Tunnel Junctions. *Nano Letters* **2021**, *21*, 175–181, PMID: 33264014.

[25] Seo, J. et al. Nearly room temperature ferromagnetism in a magnetic metal-rich van der Waals metal. *Science Advances* **2020**, *6*, eaay8912.

[26] Yang, X.; Zhou, X.; Feng, W.; Yao, Y. Strong magneto-optical effect and anomalous transport in the two-dimensional van der Waals magnets Fe_nGeTe_2 (n = 3, 4, 5). *Phys. Rev. B* **2021**, *104*, 104427.

[27] Kohn, W. Analytic Properties of Bloch Waves and Wannier Functions. *Phys. Rev.* **1959**, *115*, 809–821.

[28] Heine, V. On the General Theory of Surface States and Scattering of Electrons in Solids. *Proceedings of the Physical Society* **1963**, *81*, 300.

[29] Prodan, E. Analytic structure of Bloch functions for linear molecular chains. *Phys. Rev. B* **2006**, *73*, 035128.

[30] Reuter, M. G. A unified perspective of complex band structure: interpretations, formulations, and applications. *Journal of Physics: Condensed Matter* **2016**, *29*, 053001.

[31] Bosoni, E.; Sanvito, S. Complex band structure with non-orthogonal basis set: analytical properties and implementation in the SIESTA code. *Journal of Physics: Condensed Matter* **2021**, *34*, 105501.

[32] Kim, S.-R.; Park, I. K.; Yoo, J.-G.; Seo, J.; Kim, J.-G.; Park, J.-H.; Kim, J. S.; Kim, K.; Lee, G.; Ko, K.-T. Role Of Orbital Bond and Local Magnetism In Fe_3GeTe_2 and Fe_4GeTe_2: Implication For Ultrathin Nano Devices. *ACS Applied Nano Materials* **2022**, *5*, 10341–10347.

[33] Ghosh, S.; Ershadrad, S.; Borisov, V.; Sanyal, B. Unraveling effects of electron correlation in two-dimensional Fe_nGeTe2 (n = 3, 4, 5) by dynamical mean field theory. *npj Computational Materials* **2023**, *9*, 86.

[34] Cococcioni, M.; de Gironcoli, S. Linear response approach to the calculation of the effective interaction parameters in the LDA + U method. *Phys. Rev. B* **2005**, *71*, 035105.

[35] Janas, D. M.; Droghetti, A.; Ponzoni, S.; Cojocariu, I.; Jugovac, M.; Feyer, V.; Radonjić, M. M.; Rungger, I.; Chioncel, L.; Zamborlini, G.; Cinchetti, M. Enhancing Electron Correlation at a 3d Ferromagnetic Surface. *Advanced Materials* **2023**, *35*, 2205698.

[36] Sanvito, S.; Rocha, A. R. Molecular-Spintronics: The Art of Driving Spin Through Molecules. *Journal of Computational and Theoretical Nanoscience* **2006**, *3*, 624–642.

[37] Butler, W. H.; Zhang, X.-G.; Schulthess, T. C.; MacLaren, J. M. Spin-dependent tunneling conductance of Fe|MgO|Fe sandwiches. *Phys. Rev. B* **2001**, *63*, 054416.

[38] Li, X.; Lü, J.-T.; Zhang, J.; You, L.; Su, Y.; Tsymbal, E. Y. Spin-Dependent Transport in van der Waals Magnetic Tunnel Junctions with Fe_3GeTe_2 Electrodes. *Nano Letters* **2019**, *19*, 5133–5139, PMID: 31276417.

[39] Xie, Y.; Rungger, I.; Munira, K.; Stamenova, M.; Sanvito, S.; Ghosh, A. W. *Nanomagnetic and Spintronic Devices for Energy-Efficient Memory and Computing*; John Wiley Sons, Ltd, 2016; Chapter 4, pp 91–132.

[40] Belashchenko, K. D.; Velev, J.; Tsymbal, E. Y. Effect of interface states on spin-dependent tunneling in Fe/MgO/Fe tunnel junctions. *Phys. Rev. B* **2005**, *72*, 140404.

[41] Rungger, I.; Mryasov, O.; Sanvito, S. Resonant electronic states and $I{-}V$ curves of Fe/MgO/Fe(100) tunnel junctions. *Phys. Rev. B* **2009**, *79*, 094414.
